# Advanced Metal–Organic Framework-Based Sensor Systems for Gas and Environmental Monitoring: From Material Design to Embedded Applications

**DOI:** 10.3390/s25216539

**Published:** 2025-10-23

**Authors:** Alemayehu Kidanemariam, Sungbo Cho

**Affiliations:** 1 Department of Electronic Engineering, Gachon University, Seongnam-si 13120, Gyeonggi-do, Republic of Korea; 2 Department of Semiconductor Engineering, Gachon University, Seongnam-si 13120, Gyeonggi-do, Republic of Korea; 3 Gachon Advanced Institute for Health Science & Technology, Gachon University, Incheon 21999, Republic of Korea

**Keywords:** metal–organic frameworks (MOFs), environmental sensors, sensor systems, material design, embedded systems, air and water quality monitoring, sensor integration, edge computing, signal processing, greenhouse gases, sensor stability

## Abstract

Environmental pollution is a global issue presenting risks to ecosystems and human health through release of toxic gases, existence of volatile organic compounds (VOCs) in the environment, and heavy metal contamination of waters and soils. To effectively address this issue, reliable and real-time monitoring technology is imperative. Metal–organic frameworks (MOFs) are a disruptive set of materials with high surface area, tunable porosity, and abundant chemistry to design extremely sensitive and selective pollutant detection. This review article gives an account of recent advances towards sensor technology for MOFs with application specificity towards gas and environment monitoring. We critically examine optical, electrochemical, and resistive platforms and their interfacing with embedded electronics and edge artificial intelligence (edge-AI) to realize smart, compact, and energy-efficient monitoring tools. We also detail critical challenges such as scalability, reproducibility, long-term stability, and secure data management and underscore transforming MOF-based sensors from lab prototype to functional instruments to ensure safe coverage of human health and to bring about sustainable environmental management.

## 1. Introduction

The expansion of industrial activities, growing number of vehicles, and large-scale chemical processing appears to have added substantially to toxic gases in the environment, with adverse consequences for environmental integrity and public health [1,2,3]. Pollutants like nitrogen oxides (NO_x_), sulfur dioxide (SO_2_), carbon monoxide (CO), ozone (O_3_), and various volatile organic compounds (VOCs) do not just linger uniformly; they fluctuate across neighborhoods, cities, and regions, sometimes spiking unexpectedly [4,5,6]. Long-term exposure and occasional brief periods of high levels can cause respiratory and cardiovascular diseases, neurological effects, and environmental issues such as smog, acid rain, and imbalanced ecosystems [7,8]. While MOFs have emerged as promising materials for selective and real-time detection of these pollutants, their potential toxicity to humans and the environment should also be considered, as highlighted in recent studies [9,10]. There is clearly a growing global need for research to aid air quality management and regulation, alongside the development of safe and effective sensing technologies. There is clearly a growing global need for research to aid air quality and regulation, with a demand for more selective and real-time detection technologies to monitor these pollutants [11].

Researchers have investigated a range of sensing methods to identify toxic gases over the years, each reinforced by distinct advantages and disadvantages. Photonic methods, for instance, infrared (IR) and ultraviolet (UV) absorption or fluorescence detection, exploit light and gas molecule interaction to permit high selectivity [12,13]. While in controlled environments, these can have excellent precision, these are generally large, expensive equipment-based methods and are susceptible to interference by dust, humidity, or other environmental factors [14]. Acoustic sensors, like quartz crystal microbalance (QCM) devices, can detect minuscule mass changes upon gas adsorption and are extremely sensitive, but they are notoriously difficult to miniaturize and deploy reliably outside the lab [15]. Colorimetric or other visual indicator methods offer simple readouts, yet they tend to be qualitative, often irreversible, and ill-suited for continuous monitoring [16].

Metal oxide semiconductor (MOS) sensors, including tin dioxide (SnO_2_), zinc oxide (ZnO), and tungsten trioxide (WO_3_), have traditionally dominated gas sensing because of their affordability, structural stability, and straightforward fabrication [17,18]. Their sensing relies on the adsorption and reaction of oxygen species on the surface, a process that typically requires elevated temperatures (200–400 °C) [19]. This dependence on heat increases power consumption and makes MOS sensors less suitable for compact, portable, or wearable monitoring devices. Additionally, they can suffer from limited selectivity and cross-sensitivity, particularly in environments with complex gas mixtures or fluctuating humidity [20]. While strategies like doping or surface modification can improve performance, these adjustments rarely achieve the precise molecular recognition needed for highly selective toxic gas detection.

Electrochemical sensors have emerged as extremely promising tools for the monitoring of toxic gases, owing mainly to their capacity to present an optimal trade-off among portability, selectivity, and sensitivity [21,22]. These sensors detect changes in current, potential, or impedance that arise from redox reactions between the target gas and the electrode surface [23]. When coupled with carefully designed materials such as metal–organic frameworks (MOFs), graphene, or carbon nanotubes (CNTs), hybrid electrochemical platforms can detect gases at parts-per-billion levels while responding selectively to specific analytes [24]. Unlike MOS sensors, electrochemical systems can operate efficiently at ambient temperatures, which drastically reduces power requirements and allows integration into compact or wearable devices [25]. Furthermore, their electrical signals lend themselves well to real-time processing and predictive analysis, particularly when combined with edge-computing or AI, making them strong candidates for connected, intelligent environmental monitoring systems [26,27]. While traditional approaches remain valuable in some contexts, electrochemical sensors, especially those leveraging MOFs, appear to offer a uniquely balanced solution for next-generation gas monitoring.

Among advanced materials, MOFs stand out as particularly compelling. These crystalline, porous networks, composed of metal nodes linked by organic molecules, provide enormous surface areas, tunable pore sizes, and versatile chemical functionality [28,29]. Such properties allow MOFs to preferentially capture certain gas molecules, enhancing both sensitivity and selectivity. In addition, some MOFs can operate at room temperature, which reduces energy consumption and facilitates integration into compact devices [30]. They can also be engineered to respond through multiple sensing modes, electrical, optical, or electrochemical, depending on the target gas and application [31]. While MOFs are celebrated for their high surface area, tunable porosity, and modular functionality, their long-term structural and chemical stability remains a critical challenge for sensor applications [32]. Inherent frameworks may degrade under humid or chemically harsh conditions, potentially compromising device performance [33]. Strategies such as post-synthetic functionalization, defect engineering, hybridization with polymers or carbon-based materials, and composite formation have been developed to enhance stability while maintaining sensitivity and selectivity, making MOF-based sensors more viable for real-world deployment.

What makes MOFs especially interesting is their modularity. Post-synthetic modifications, functionalization, or hybridization with nanomaterials such as graphene or CNTs can fine-tune their performance for particular gases [34]. This adaptability makes MOFs promising candidates for wearable devices or small environmental monitors. There is also growing work on coupling MOF sensors with microelectronics and edge-AI systems, which enables real-time analysis, pattern recognition, and predictive modeling [35]. In principle, such integration could support smarter monitoring networks, offering early warnings, continuous air quality feedback, and adaptive sensing in dynamic environments [36].

Of course, significant challenges remain before MOF-based sensors can be widely deployed [37]. Issues such as reproducibility at scale, long-term stability, humidity tolerance, and secure data handling need careful attention [38]. Optimizing interfaces between MOFs and device components, alongside standardized fabrication protocols, will likely be critical for broader adoption [39]. Nevertheless, the combination of MOFs’ intrinsic sensing capabilities with miniaturized electronics and AI-driven analytics suggests a promising path toward next-generation toxic gas monitoring systems [40].

In this review, we explore the landscape of MOF-based toxic gas sensors, with an emphasis on design strategies that enhance sensitivity and selectivity, the variety of sensing platforms developed, and their integration into intelligent, compact devices. We also consider how MOFs might shape the future of portable, low-power, AI-enabled environmental monitoring tools that can protect human health while supporting sustainable environmental management.

## 2. Toxic Gases: Sources, Effects, and Monitoring Needs

**Figure 1:**
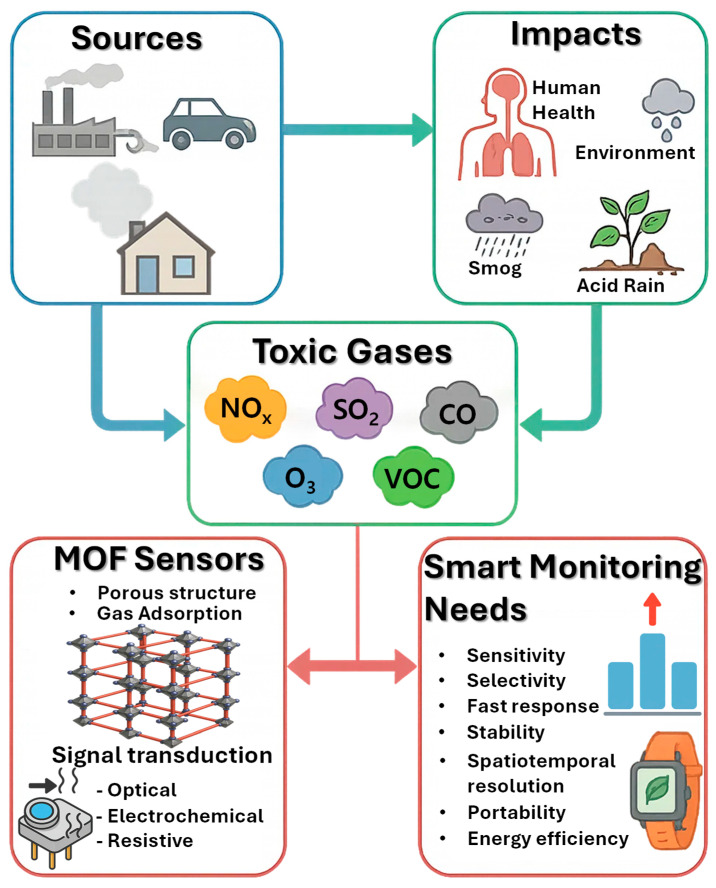
Origin of toxic gases, associated environmental effects, and possible detection and mitigation strategies using sensors.

Toxic gases in the environment are a pressing concern due to their widespread presence, variable concentrations, and potentially severe effects on human health and ecosystems (Figure 1) [41]. Unlike particulate pollutants, gases can disperse over large areas, accumulate under certain meteorological conditions, and react with other atmospheric components to form secondary pollutants such as smog or acid rain. Monitoring these gases is therefore not just a matter of detecting their presence but also understanding their dynamics in space and time, assessing exposure risks, and designing strategies to mitigate their impacts [42]. The diverse sources, chemical properties, and environmental interactions of these gases pose distinct challenges for detection technologies, requiring sensors that are sensitive, selective, reliable, and adaptable to real-world conditions.

### 2.1. Major Toxic Gases in the Environment

#### 2.1.1. Toxic Gas Sources

Toxic gases in the environment originate from a complex interplay of anthropogenic and natural sources, often overlapping in ways that complicate their mitigation. Industrial activities such as power generation, metal processing, and chemical manufacturing are primary contributors, releasing significant quantities of nitrogen oxides (NO_x_), sulfur dioxide (SO_2_), and volatile organic compounds (VOCs) [43,44]. In urban areas, vehicular emissions further elevate the levels of NO_x_, carbon monoxide (CO), and VOCs. Agricultural practices also play a role in fertilizer application and livestock management emit ammonia and other reactive gases, while chemical and petrochemical industries continuously discharge a diverse mixture of atmospheric pollutants [45].

Among these, NO_x_, SO_2_, CO, ground-level O_3_, and VOCs stand out because of their far-reaching effects on both ecosystems and human health. NO_x_, mostly from cars, factories, and power plants, is not just invisible chemicals floating in the air; it quietly affects the world around us [46]. It can acidify soil and waterways, weaken crops and forests, create smog that blankets our cities, and even stress wildlife. People feel it too, with worsened asthma and breathing problems, showing just how closely our health and the environment are connected.

Similarly, SO_2_ released mainly from fossil fuel combustion and industrial operations readily reacts with atmospheric moisture to form acid rain, degrading soil fertility, damaging vegetation, and altering aquatic ecosystems [47]. Even moderate increases in SO_2_ can stress plant communities and diminish biodiversity, emphasizing its long-term ecological impact.

CO, a colorless and odorless gas predominantly emitted by incomplete combustion in vehicles and industries, disrupts oxygen transport in the bloodstream, posing acute toxicological risks [48]. Although microbial CO oxidation contributes marginally to its environmental balance, persistent emissions remain hazardous. Ground-level ozone (O_3_), formed through photochemical reactions involving NO_x_ and VOCs, is another critical air pollutant that causes lung irritation, reduces crop yields, and accelerates material degradation [49].

VOCs, emitted from solvents, paints, fuel combustion, and industrial processes, encompass numerous compounds such as benzene, formaldehyde, vinyl chloride, and 1,3-butadiene, many of which are confirmed human carcinogens [50]. These compounds participate in photochemical reactions leading to smog formation and exert both acute and chronic effects on human health, including respiratory irritation, neurological disorders, and cancer. Therefore, stringent monitoring and control of VOCs and toxic gases are essential for ensuring environmental and public safety.

#### 2.1.2. Health and Environmental Impacts

The health effects of toxic gases are often insidious, developing gradually through long-term exposure. Chronic inhalation can increase the risk of respiratory illnesses, cardiovascular diseases, neurological impairments, and various cancers [51]. Environmentally, these pollutants drive the formation of smog and acid rain, disrupt soil and water chemistry, and threaten plant and aquatic life. Gaseous precursors such as VOCs and NO_x_ also contribute to secondary pollutants like ozone and fine particulate matter (PM_2.5_), amplifying their overall impact.

To emphasize their real-world significance, the legally permitted indoor concentration limits and health impacts of major toxic gases and VOCs are summarized in Table 1 [52,53,54,55]. These reference values, defined by international organizations such as WHO, EPA, and the EU, serve as important benchmarks for assessing air quality and designing efficient sensing materials.

Globally, the extent of human and ecological damage depends on environmental conditions, socioeconomic factors, and pre-existing vulnerabilities [56]. Prolonged exposure can lead to ecosystem degradation, soil contamination, and even social inequities, where marginalized communities face disproportionate exposure to pollutants.

Tackling this problem is not simple. We probably need a mix of approaches, better monitoring systems, some form of ecological restoration, stricter enforcement of environmental regulations, and, perhaps surprisingly, new materials like MOFs [57]. These MOFs seem promising for trapping industrial pollutants, from SO_2_ and NO_2_ to NH_3_, H_2_S, CO, VOCs, radioactive gases, and even mercury vapor, which could help protect both people and the environment. While no single solution is perfect, combining these strategies seems like the best way to reduce the health and ecological toll of toxic gas emissions [58].

### 2.2. Monitoring Requirements and Challenges

Monitoring toxic gases is becoming increasingly important as safety concerns grow and evidence mounts about their impact on both human health and the environment. Gases like NO_2_, CO, CO_2_, methane, butane, hydrogen sulfide (H_2_S), and propane aren’t just unpleasant; prolonged exposure can lead to respiratory illnesses, cardiovascular issues, and, in some cases, neurological damage [59]. On top of that, these pollutants can quietly disrupt ecosystems by degrading soil quality, contaminating water sources, and threatening biodiversity. All this suggests that having reliable, real-time detection systems isn’t just useful; it is becoming essential.

Of course, building an effective monitoring system is not straightforward. It needs to check several boxes at once: high sensitivity to catch trace amounts before they hit dangerous thresholds, good selectivity so it is not thrown off by other gases in the air, fast response times to capture sudden spikes, and long-term stability to keep running accurately in different environmental conditions [60]. There has been progress. With advances in sensor design and IoT integration, we now have automated systems that can detect hazardous gas concentrations in real time and immediately trigger countermeasures, anything from sounding alarms to switching on exhaust fans or activating ventilation systems.

That said, the challenges are far from solved. A surprising number of facilities still rely on manual or outdated safety setups, which can slow emergency responses when seconds matter [61]. Cost is another sticking point, especially for small and medium-sized industries trying to balance safety with limited budgets. And even when the hardware is in place, issues like proper calibration, regular maintenance, and integrating new systems with old infrastructure can make deployment tricky.

In the end, effective toxic gas monitoring seems to require a blend of reliable technology, affordability, and seamless integration. It is not just about detecting danger but doing so early enough and accurately enough to prevent harm. Getting this right matters not only for protecting human health but also for maintaining environmental quality and keeping industrial spaces genuinely safe.

#### 2.2.1. Sensitivity, Selectivity, Response Time, Stability

In practice, no single sensor technology perfectly satisfies all these requirements, which is why advanced materials, such as MOFs or nanostructured electrodes, are increasingly explored [62]. A sensor’s ability to reliably discriminate between gases, maintain performance under humidity fluctuations, and respond quickly to concentration changes can be the difference between effective early warning systems and unreliable data [63].

The performance of gas sensors depends strongly on sensitivity and selectivity. High sensitivity enables the detection of trace gas concentrations, while high selectivity ensures accurate identification of target gases even in the presence of interfering species. Without these traits, sensors risk delayed detection or false readings, reducing reliability [64]. Optimizing surface modification techniques such as catalyst decoration, composite formation, and surface functionalization, along with selecting suitable receptor materials, is essential to achieve both. Balancing sensitivity and selectivity enhances detection accuracy, enabling efficient monitoring in applications like air pollution control, hazardous gas detection, food quality assessment, and personal health monitoring [65].

**Figure 2:**
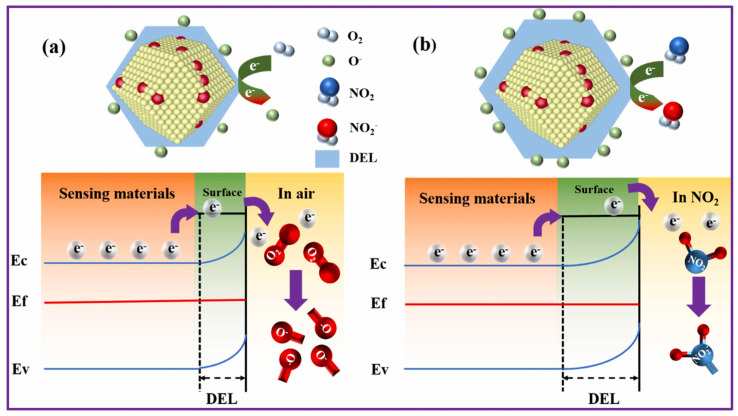
Proposed NO_2_ sensing mechanism of 2% La-doped ZnO (ZL-2) porous nanocages: (**a**) behavior in ambient air, where La^3+^ substitution increases carrier density and shifts the Fermi level, and (**b**) interaction with NO_2_ molecules, showing enhanced adsorption and charge transfer due to the synergistic effect between La_2_O_3_ and ZnO facilitated by the nanoporous structure. Reproduced with permission from Elsevier [66].

Hussain et al. reported a lanthanum (La)-doped ZnO porous nanocages synthesized using a relatively simple one-step co-precipitation followed by MOF encapsulation and calcination [66]. Interestingly, while several doping levels were tested (2%, 4%, and 8%), the 2% La-doped sample consistently gave the best results. It showed a strong response of 118 at 50 ppm NO_2_, reacted in just 28 s, and managed to detect concentrations as low as 5.68 ppb. What’s more, it held up reasonably well under humid conditions, displayed good reproducibility, and, perhaps most importantly, was highly selective toward NO_2_ over other gases. Figure 2 offers a visual summary of the proposed sensing mechanism. In simple terms, part of the Zn^2+^ ions in the ZnO lattice are replaced by La^3+^, and because La behaves as a donor impurity, it pushes extra carriers into the conduction band. This appears to shift the Fermi level upward, altering the electronic landscape in a way that favors NO_2_ detection. Combine that with the nanoporous structure, which provides plenty of sites for NO_2_ to latch onto, and you get a synergistic effect between La_2_O_3_ and ZnO that boosts both adsorption and charge transfer.

#### 2.2.2. Portability and Energy Efficiency

For widespread environmental monitoring, especially in urban or remote settings, sensors must be compact, portable, and energy-efficient [67]. Fixed monitoring stations provide valuable data but are costly and lack fine-scale resolution. Wearable devices, drone-mounted sensors, or low-power IoT-enabled platforms can fill these gaps, provided the sensing material and electronics can operate reliably at ambient conditions without excessive power consumption.

Hydrazine (N_2_H_4_) is a highly reactive chemical widely used in industry, but it also carries significant environmental and health risks, which makes rapid detection a pressing concern. Li et al. recently reported a ratiometric fluorescent sensor, DIPOT, that was designed for ultra-sensitive and portable monitoring of N_2_H_4_ in environmental settings [68]. The sensor exhibits an impressively low detection limit of 4.5 nM and a noticeable 156 nm blue shift, changing fluorescence from red to green and allowing for easy visual identification. What makes DIPOT particularly appealing is its integration into portable test strips that can be read via a smartphone app, enabling on-site detection of both N_2_H_4_ vapors and solutions. The system was tested across 20 diverse environmental samples, ranging from water and soil to crops and food, showing consistent and reliable performance. Taken together, this approach suggests a practical pathway toward real-time, field-deployable detection of toxic gases, though further testing in more complex or industrial environments would be needed to fully validate its robustness.

In a similar study, portable gas sensors are increasingly relied upon for environmental and health monitoring, but their design must balance efficiency with safety (Table 2) [69]. Encapsulated sensors made with metallic or conductive inks appear to offer a promising solution, detecting toxic gases at very low concentrations down to 607 ppb in studies of Ag^+^ leaching while significantly reducing the cytotoxic effects observed in non-encapsulated devices. By limiting ion release, encapsulation not only enhances biocompatibility but also improves stability, making the sensors more reliable and responsive in practical, real-world settings.

## 3. Sensor Materials for Toxic Gas Detection

Over the past few decades, gas-sensing technologies have undergone remarkable progress to meet the growing demand for highly sensitive, selective, and stable platforms for detecting toxic gases in complex environments [70,71]. Conventional sensing materials, including electrochemical probes, metal oxides, and optical materials, have laid the groundwork for modern gas detection systems. These materials remain indispensable in industrial, environmental, and safety applications because of their technological maturity, ease of fabrication, and commercial availability [72,73]. However, despite their advantages, traditional sensors face limitations such as cross-sensitivity to non-target gases, humidity interference, high power requirements, and reduced long-term stability [74]. These challenges have driven the exploration of advanced nanomaterials and hybrid composites that provide tunable surface chemistry, enhanced detection limits, and improved compatibility with portable or embedded platforms.

### 3.1. Traditional Sensor Materials

#### 3.1.1. Metal Oxides and Chemiresistive Materials

Metal oxide semiconductors (MOS), such as SnO_2_, ZnO, and TiO_2_, represent one of the most extensively studied families of sensing materials owing to their robustness and wide applicability for toxic gas detection [75]. Their operation is based on resistance modulation resulting from the adsorption and reaction of analyte molecules on heated oxide surfaces. While MOS sensors exhibit fast response and high sensitivity, they typically require elevated operating temperatures, leading to high power consumption and limited long-term stability [76]. Selectivity also remains a challenge since many gases produce similar resistance changes, complicating detection in mixed environments.

Recent advances have focused on defect engineering and nanostructuring to improve sensitivity and selectivity. For instance, Borse et al. demonstrated that controlled Sn doping in LaCoO_3_ perovskite structures significantly improved sensitivity toward CO_2_, NH_3_, and NO_2_ by tuning oxygen vacancies and surface reactivity [77]. Similarly, Au@In_2_O_3_ hollow-sphere arrays fabricated via template-assisted magnetron sputtering exhibited excellent responses to n-butanol (R_a_/R_g_ = 1054 at 325 °C) due to their hollow structure, Au catalytic effects, and metal–semiconductor junction formation [78].

The integration of nanostructured hybrids such as metal oxide/graphene or metal oxide/TMDC composites has emerged as a promising route to enhance chemiresistive performance through synergistic catalytic activity and improved charge transport [79]. These hybrid materials provide flexibility, reduced weight, and enhanced adaptability to varying environments, paving the way for next-generation chemiresistive gas sensors. However, scalability, reproducibility, and stability under fluctuating conditions remain challenges that limit large-scale deployment [80].

#### 3.1.2. Optical and Photonic Materials

Optical gas sensors utilize the interaction of light with matter, relying on absorption, emission, or scattering processes to detect specific gases [14]. IR and UV absorption-based sensors are particularly effective for monitoring greenhouse gases such as CO_2_, CH_4_, and VOCs [81]. Fluorescence-based sensors, meanwhile, offer high sensitivity and real-time detection capabilities [82]. Recently, Chen et al. reported that a multicomponent optical biosensor was demonstrated using hollow-core fiber (HCF) photothermal spectroscopy integrated with frequency-division multiplexing (FDM) [82]. The system employs a single antiresonant HCF (AR-HCF) as the sensing chamber, enabling broadband light transmission from the near-infrared (NIR) to the mid-infrared (MIR) region and covering distinct absorption lines of water vapor (H_2_O, 1.39 μm), CO_2_, 2.00 μm, and CO, 4.60 μm. Multiple pump lasers at these wavelengths are modulated at separate frequencies and coupled into the HCF, generating photothermal phase shifts that are detected by a common Fabry–Perot interferometer probe at 1.55 μm. Using harmonic demodulation, the sensor achieved simultaneous and highly sensitive detection of the three gases, with limits of detection (LODs) of 2.7 ppm for H_2_O, 25 ppb for CO_2_, and 9 ppb for CO within a 1 s integration time. Remarkably, with extended averaging (1000 s), the detection limits improved to 222 ppb, 1.5 ppb, and 0.6 ppb, respectively. The optical biosensor demonstrated strong stability, with less than 1.7% signal fluctuation over 2 h, and effective calibration of humidity interference using the H_2_O reference signal. These results highlight the potential of HCF-based photothermal spectroscopy for ultra-sensitive, multiplexed gas biosensing applications.

Moreover, a fluorescence-based optical biosensor was developed for the visual detection of H_2_S, enabling real-time monitoring of meat spoilage. The sensing platform utilized ratiometric fluorescence from copper nanoclusters (CuNCs) and nitrogen-doped carbon quantum dots (CNQDs), which provided dual-emission signals for reliable detection [83]. The fluorescence intensity ratio exhibited a clear decrease with increasing sulfide concentration over the range of 0–3 μM, achieving a detection limit of 62.7 nM in solution. To enable practical gas-phase sensing, the ratiometric fluorescent probes were immobilized on a paper substrate for direct capture of H_2_S in air. Under UV illumination, RGB image processing allowed visual quantification of H_2_S with a remarkably low detection limit of 4.35 ppt across the 0–45.2 ppt concentration range. This portable and user-friendly optical biosensor not only simplifies the detection process but also ensures high sensitivity and stability, offering a promising strategy for rapid and reliable monitoring of H_2_S during food storage and spoilage assessment.

**Figure 3:**
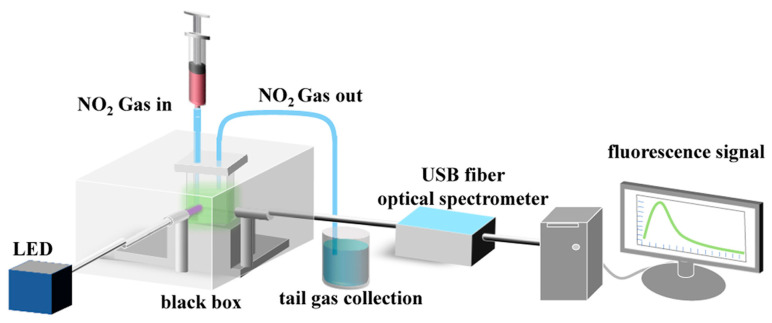
Schematic representation of the fluorescence-based detection mechanism of NO_2_ using engineered g-C_3_N_4_ nanobelts. Thermal treatment and alkaline hydrolysis yield nanobelts with enhanced fluorescence emission (494 nm, 23.6% QY). Reproduced with permission from Elsevier [84].

In another study, graphitic carbon nitride (g-C_3_N_4_) nanostructures typically exhibit blue-shifted emission compared to their bulk counterparts due to quantum confinement effects, which can limit their effectiveness in bioimaging and sensing [84]. To overcome this limitation, a novel strategy was developed to engineer g-C_3_N_4_ nanomaterials with tunable fluorescence wavelength and enhanced intensity. Bulk g-C_3_N_4_ was subjected to controlled high-temperature treatment (750 °C, 2 h), followed by hydrolysis in alkaline solution (4 mol L^−1^ NaOH, 8 h), yielding highly fluorescent g-C_3_N_4_ nanobelts with an emission peak at 494 nm and a quantum yield of 23.6%, representing a significant improvement over bulk material. Leveraging these nanobelts as fluorescent probes, a portable optical gas sensor was fabricated for the reversible detection of toxic NO_2_ at room temperature. As shown in Figure 3, upon exposure to NO_2_ gas at room temperature, adsorption of NO_2_ molecules induces electron-withdrawing interactions that quench the fluorescence signal. The reversible adsorption–desorption process enables sensitive and selective detection of NO_2_ under ambient conditions, avoiding the high operating temperatures and humidity interference typically associated with chemiresistive sensors. Unlike conventional chemiresistive gas sensors that require elevated operating temperatures and are susceptible to humidity interference, the fluorescence-based platform enabled stable, sensitive, and selective NO_2_ sensing under ambient conditions, highlighting its potential for practical environmental monitoring applications.

In similar studies, sensor active materials were incorporated by deposition over the other materials for advanced or effective gas sensor performance. In a recent study, an optical fiber-based gas sensor for ammonia detection was realized by directly depositing semiconductor oxide nanomaterials onto the fiber substrate [85]. Hexagonal MoO_3_ (h-MoO_3_) nanorods were grown on the tapered region of an optical fiber through a simple chemical bath deposition process, creating an integrated optical sensing element. A subsequent annealing treatment was employed to tune the oxidation state of h-MoO_3_, leading to significant enhancements in sensor performance. The annealed h-MoO_3_ nanorods demonstrated superior sensitivity, faster response/recovery dynamics, and improved stability compared to untreated samples. In particular, annealing at 150 °C yielded strong and reproducible room-temperature absorbance responses (0.05–0.35) with a rapid response time of 210 s toward 500 ppm NH_3_. The sensing mechanism was attributed to changes in refractive index and absorption coefficient induced by chemisorbed oxygen species and physisorbed NH_3_, which modulated the optical signal. These findings highlight that controlled nanorod deposition onto optical fibers, combined with annealing-driven defect engineering, offers a powerful strategy to enhance the performance of optical gas sensors and points toward new directions in fiber-integrated sensing technologies.

Despite their high selectivity and immunity to electromagnetic interference, optical sensors often involve costly and bulky instrumentation, limiting their widespread use in portable or wearable platforms.

#### 3.1.3. Electrochemical and Hybrid Nanomaterials

Electrochemical sensors rely on redox reactions between target analytes and electrode surfaces, converting chemical interactions into measurable electrical signals [86]. They are widely used for detecting toxic gases such as CO, NO_2_, and SO_2_ owing to their high sensitivity, low detection thresholds, and minimal power consumption.

**Figure 4:**
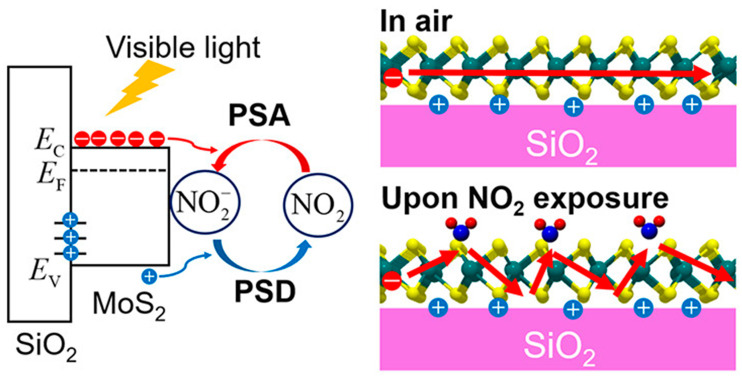
Schematic illustration of the electrochemical mechanism of NO_2_ gas sensing on a MoS_2_-based field-effect transistor (FET) sensor. Upon illumination, photoexcited charge carriers are generated in the MoS_2_ channel. Reproduced with permission from ACS [87].

Recent advances have demonstrated how integrating photoactivation can further improve electrochemical sensing. Tabata et al. showed that MoS_2_-based field-effect transistor (FET) sensors exhibit strong NO_2_ sensitivity under visible-light illumination, enabling low-power operation compared to conventional thermally activated systems [87]. As illustrated in Figure 4, NO_2_ adsorption on the MoS_2_ surface withdraws electrons, decreases carrier mobility, and reduces channel conductance. This process follows the Langmuir isotherm model, ensuring reversible sensing behavior with detection limits in the low parts per billion (ppb) range. The linear improvement of response and recovery rates with increasing light intensity underscores the potential of photostimulated electrochemical sensors for real-time toxic gas monitoring.

Further progress has been made by incorporating nanometals into 2D materials. In one study, MoS_2_ monolayers grown via chemical vapor deposition were functionalized with gold nanoparticles (AuNPs) through microwave-assisted absorption [88]. The optimized low-temperature (~120 °C) and rapid (~1 min) functionalization process resulted in a device with a clear threshold voltage shift (~0.5 V) and a sensing response of ~10% upon exposure to 200 ppb NO_2_ at near-room temperature (30 °C). Importantly, the sensor demonstrated an ultralow theoretical detection limit of 0.183 ppb, surpassing many previously reported MoS_2_-based gas sensors. Functionalization with Au NPs not only enhanced sensitivity but also improved selectivity toward NO_2_ in complex gas environments such as exhaled breath, while maintaining excellent stability under varying humidity and temperature conditions. Furthermore, UV irradiation was shown to accelerate adsorption–desorption kinetics, thereby reducing recovery times to only a few seconds. These findings highlight the critical role of nanometal functionalization and photoactivation in advancing electrochemical gas sensors with ppb-level detection limits for health-monitoring applications.

Despite these achievements, the traditional electrochemical materials suffered from low performance due to electrode fouling, electrolyte evaporation, and cross-interference from other gases. Despite these drawbacks, electrochemical sensors remain widely adopted in portable detectors and wearable monitoring devices owing to their compact size and relatively low cost.

Moreover, although various conventional sensing materials, including metal oxides, conducting polymers, and carbon-based nanomaterials, have been extensively employed for toxic gas detection, they commonly face intrinsic challenges such as high operating temperatures, limited selectivity, and instability under fluctuating environmental conditions [89]. These shortcomings hinder their broader applicability, especially for detecting trace or low-concentration gases. To address these issues, porous crystalline materials like MOFs have emerged as promising alternatives owing to their exceptionally high surface area, tunable pore architectures, and versatile functionalization capabilities, making them highly attractive candidates for next-generation toxic gas sensors.

## 4. Metal–Organic Frameworks (MOFs) in Gas Sensing

MOFs have emerged as next-generation gas sensing materials due to their crystallinity, high porosity, and exceptional chemical tunability [90]. Unlike conventional sensing platforms, MOFs provide a modular architecture where pore size, shape, and surface chemistry can be precisely tailored to enhance sensitivity, selectivity, and stability [91]. Their ordered pore networks promote efficient gas adsorption and enable specific host–guest interactions crucial for signal transduction. Advances in MOF composites and hybrids with conductive nanomaterials further boost performance, leading to sensors with rapid response, robustness, and real-world applicability [24]. By integrating tunable functionalities such as redox-active or photoresponsive sites, MOFs extend beyond traditional porous materials and position themselves as versatile candidates for smart, high-performance gas sensor technologies.

### 4.1. Fundamental Properties of MOFs Relevant to Sensing

#### 4.1.1. Porosity, High Surface Area, Tunable Chemical Functionality

The hallmark of MOFs lies in their exceptionally high surface area and porosity, which provide abundant active sites for gas adsorption and interaction [92]. Their tunable pore sizes and chemical functionalities allow selective binding of target gas molecules, leading to enhanced sensitivity and discrimination, even at trace concentrations.

Lee et al. Functionalization and noble metal incorporation in MOFs are effective strategies to improve their porosity, surface area, and sensing capabilities [93]. In a recent example, a sulfone-functionalized Zr-MOF (Zr–BPDC–SO_2_) and its Pd-embedded composite were synthesized through tailored functional group modifications. Structural and surface analyses confirmed that functionalization not only preserved the crystalline integrity but also improved accessible porosity and active surface sites, creating stronger host–guest interactions with target gases. These modifications enhanced ethanol sensing through hydrogen bonding between sulfonyl groups and ethanol molecules, while Pd incorporation further boosted hydrogen sensing by catalytically facilitating adsorption and charge transfer. The combined effects of functionalization and metal embedding highlight how tuning MOF porosity and surface chemistry can directly translate into superior sensitivity, selectivity, and dynamic response for gas monitoring applications.

In another example, UiO-66 frameworks functionalized with nitrogen-containing groups (−NH_2_, −NO_2_) and blended with conjugated polymers formed hybrid sensing films with significantly improved porosity and interfacial interactions [94]. The introduced functional groups enhanced MOF porosity and provided abundant adsorption sites for target gases, improving interfacial interactions within the composite. As a result, amine-functionalized UiO-66 blended with poly(3-hexylthiophene) (P3HT) demonstrated a twofold increase in sensitivity (≈2%/ppm) and an ultralow detection limit of 0.001 ppt, with outstanding selectivity toward NO_2_ over interfering gases such as CO_2_ and SO_2_. These results highlight how functionalization-driven improvements in porosity and surface chemistry of MOFs can significantly elevate hybrid sensor performance.

#### 4.1.2. Modular Design and Post-Synthetic Modification

MOFs can be rationally designed using different combinations of metal nodes and organic linkers, enabling control over pore geometry, chemical affinity, and stability. Post-synthetic modifications further extend their versatility, allowing the introduction of catalytic, luminescent, or redox-active groups that directly enhance sensing performance.

Zhang et al. reported gas sensor developed via modular MOF design combined with post-synthetic transformation can enhance sensing performance [95]. By using Pt@ZIF-8 as a precursor, MOF-derived Pt@ZnO porous nanofibers were fabricated, integrating the advantages of MOFs’ tunable frameworks with the structural stability of nanofibers. The post-synthetic incorporation of ultra-small Pt clusters (~2 nm) into the ZnO matrix prevented aggregation, increased active sites, and introduced catalytic spillover effects. These modular and post-synthetic design features enabled superior sensing of acetone, showing a 12.2-fold higher response at 20 ppm with ultrafast dynamics (2 s/5 s) compared to conventional ZnO nanocubes. This approach highlights how MOF modularity and post-synthetic modification can yield tailored architectures with optimized porosity, stability, and catalytic activity for high-performance gas sensing.

Building on this concept, linker functionalization provides another effective pathway for enhancing MOF sensing behavior. For instance, an amine-functionalized zinc-based MOF (Zn-BDC-NH_2_), synthesized via a solvothermal route, introduced −NH_2_ groups into Zn_4_O secondary building units, increasing pore size (~13 Å) and surface area (880 m^2^ g^−1^) [96]. This functionalization not only tailors the chemical environment for H_2_ adsorption but also enables selective host–guest interactions at low temperatures. As a result, Zn-BDC-NH_2_ demonstrates excellent sensing capability for low H_2_ concentrations (1–10 ppm) with high sensitivity (response value ~2.93 at 10 ppm), rapid recovery, and stable performance at just 50 °C. These results underscore how modular MOF design combined with targeted functionalization can deliver efficient, low-power gas sensors with strong potential for practical applications.

Beyond improving sensitivity and energy efficiency, modular MOF design also enables application-specific solutions. A notable example is NH_2_-MIL-101(Cr), which was integrated into a QCM sensor for the detection of HF gas released from electrolyte leakage in new energy vehicles [97]. This functionalization, combined with the intrinsic high surface area and porosity of the MOF, provided abundant active sites for gas adsorption, resulting in excellent sensitivity (LOD 500 ppb), fast response/recovery, and good reproducibility. Computational analysis further confirmed that the sensing mechanism is governed by reversible chemical adsorption, consistent with experimental findings. These results highlight how modular MOF design and targeted functionalization can deliver high-performance, application-specific gas sensors for critical safety monitoring in new energy vehicles.

**Figure 5:**
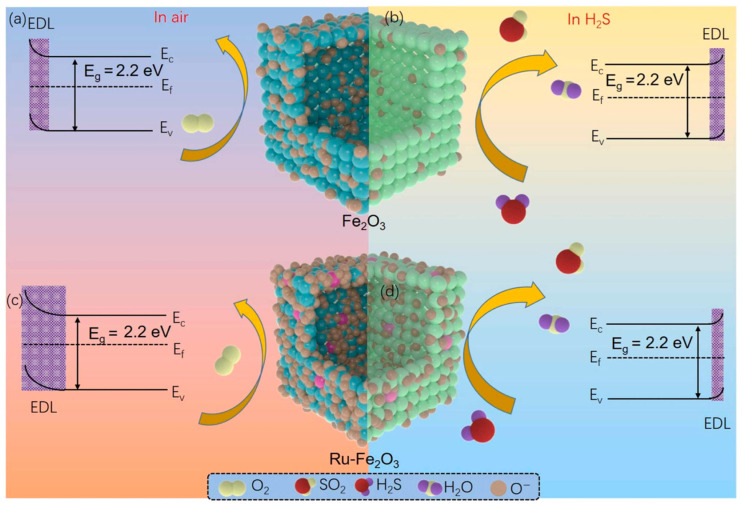
Schematic of the gas-sensing mechanism of Fe_2_O_3_ and Ru-Fe_2_O_3_ hollow-box sensors. (**a**,**c**) In air, oxygen adsorbs and ionizes on the surface, capturing electrons and forming an electron depletion layer (EDL), increasing resistance. (**b**,**d**) Exposure to reducing gases reacts with adsorbed oxygen, releasing electrons, thinning the EDL, and decreasing resistance. Reproduced with permission from Elsevier [98].

In addition to pristine MOFs, the principles of modular design and functionalization can be extended to MOF-derived materials. For example, Wang et al. reported that a hierarchical Ru-doped Fe_2_O_3_ nanobox was synthesized via a simple hydrothermal-annealing method, forming hollow and porous structures composed of small Fe_2_O_3_ nanoparticles [98]. Ru doping served as a functional modification, enhancing surface defects, oxygen vacancies, and controlling crystal size, which collectively increased the material’s active sites and gas adsorption efficiency. Figure 5 shows the sensing process, which is governed by changes in carrier concentration due to reactions between target gas molecules and adsorbed oxygen species on the material surface. In air, oxygen molecules adsorb and ionize (O^−^, O_2_^−^, O^2−^) by capturing electrons from the conduction band, forming a thick electron depletion layer (EDL) and increasing resistance (a). Upon exposure to reducing gases, the target molecules react with chemisorbed oxygen, releasing electrons back to the conduction band, thinning the EDL, and lowering resistance (b). The schematic illustrates the sequential adsorption, reaction, and carrier modulation underlying the sensor’s response. Consequently, the Ru-Fe_2_O_3_ sensor exhibited a significantly higher response to 100 ppm H_2_S (32.6), faster response/recovery times (8/34 s), and could detect ppb-level H_2_S with excellent selectivity, repeatability, and long-term stability compared to pure Fe_2_O_3_. The hierarchical porous architecture, combined with Ru-induced electronic and structural modifications, underpinned these improvements, demonstrating how targeted functionalization can elevate the performance of chemiresistive gas sensors.

Together, these examples demonstrate that modular MOF design, whether achieved through linker selection, functional group incorporation, or post-synthetic modification, provides a powerful strategy for tailoring sensing materials. Moreover, extending these design concepts to MOF-derived composites further broadens the scope of high-performance sensors, enabling not only general gas detection but also application-specific solutions.

**Figure 6:**
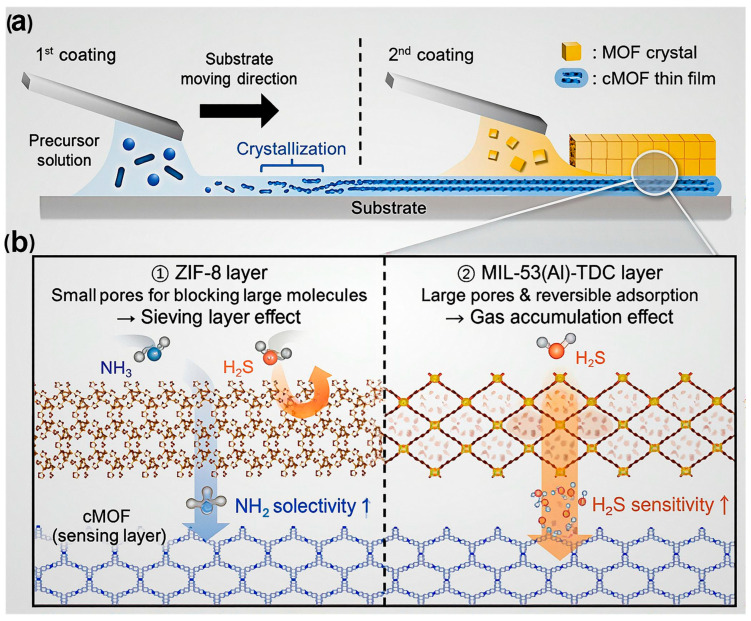
Schematic illustration of (**a**) the stepwise fabrication of layered MOF-on-cMOF films via the solution-shearing technique and (**b**) the synergistic roles of the conductive and porous MOF layers in enhancing gas adsorption, charge transport, and overall sensing performance. Reproduced with permission from ACS [99].

Moreover, MOFs’ limited electrical conductivity has historically hindered their use in chemiresistive sensors. The emergence of electrically conductive MOFs (cMOFs) has overcome this barrier, enabling efficient charge transport and enhancing sensor responsiveness. As illustrated in Figure 6, integrating cMOFs with conventional insulating MOFs in heterostructured or layered architectures allows fine-tuning of gas-sensing behavior through controlled gas–framework interactions [99]. For example, MOF-on-cMOF configurations fabricated via the solution-shearing technique exhibit enhanced sensitivity, selectivity, and rapid response/recovery toward analytes such as NH_3_, H_2_S, and NO_2_. These performance improvements arise from the synergistic effects between the conductive backbone of cMOFs and the adsorption selectivity of secondary MOF layers. Such findings highlight the growing importance of conductive MOFs in developing next-generation chemiresistive gas sensors with rationally engineered structure–property relationships.

Additionally, a core–shell composite integrating a 2D conductive MOF with a 3D porous MOF was designed to exploit synergistic interfacial effects for efficient H_2_S detection. The resulting 2D-MOF@3D-MOF heterostructure exhibited exceptional sensing performance, including an ultralow detection limit of 1.4 ppb, high sensitivity (ΔR/R_0_ = 3.37), and remarkable selectivity at room temperature [100]. These enhancements are attributed to improved charge transport pathways provided by the conductive MOF core, coupled with the high adsorption capacity of the porous shell. Mechanistic analyses further revealed that interfacial charge transfer, structural deformation, and the generation of reactive sites collectively drive the enhanced chemiresistive response. This study demonstrates the crucial role of conductive MOFs in achieving sensitive, selective, and room-temperature gas sensing.

Building upon these findings, understanding the electrical or resistive transduction mechanisms underlying such conductive MOF-based systems becomes essential, as they directly govern the signal generation process and ultimately dictate the sensitivity, selectivity, and response dynamics of MOF-based gas sensors.

### 4.2. MOF-Based Transduction Mechanisms

#### 4.2.1. Electrical/Resistive Sensing

In resistive sensing, changes in electrical conductivity arise when adsorbed gases interact with the MOF or conductive MOF composites [101]. While pristine MOFs often show limited conductivity, combining them with conductive additives (e.g., graphene, CNTs) significantly enhances their utility in chemiresistive sensors.

An early demonstration involved Cu-MOF-based sensors for acetone and NO_2_ detection, where hydrothermally synthesized Cu-MOF powders were deposited on ceramic platforms with Pt electrodes [102]. The Cu-MOF was synthesized via a hydrothermal method using benzene-1,2,4,5-tetracarboxylate linkers. Thick films of Cu-MOF powders were deposited on ceramic platforms with interdigitated Pt electrodes and integrated heaters to fabricate the sensors. Heat treatment at different temperatures yielded two devices, Cu-MOF250 and Cu-MOF400, with distinct chemiresistive behaviors: Cu-MOF400 showed enhanced acetone detection at 250 °C, while Cu-MOF250 exhibited superior NO_2_ sensing at 40 °C. Notably, Cu-MOF250 demonstrated dual sensing capability, selectively detecting acetone at 200 °C and NO_2_ near ambient temperature. The chemiresistive sensing mechanism is driven by adsorption of gas molecules onto the MOF, which modulates charge carrier density and alters the material’s electrical resistance, emphasizing the role of surface chemistry and porosity in gas detection.

**Figure 7:**
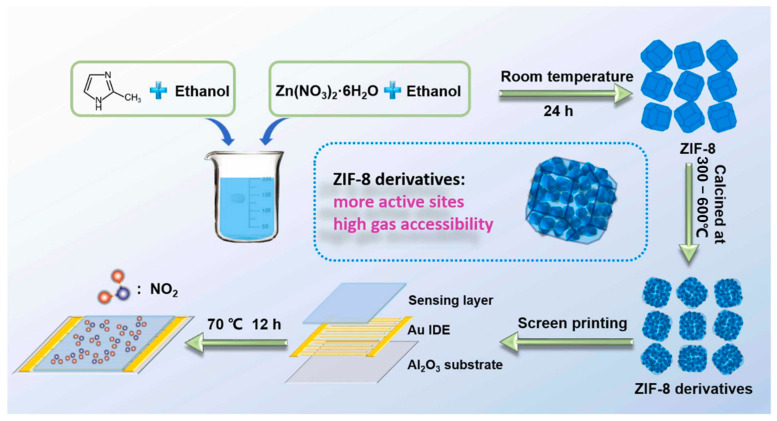
Synthesis of ZIF-8 and its derivatives. ZIF-8 was obtained from Zn(NO_3_)_2_·6H_2_O and DMF in ethanol, aged 12 h, washed, and dried at 60 °C. The precursor was pyrolyzed at 300–600 °C to yield derivatives labeled ZIF-8–300 to ZIF-8–600. Reproduced with permission from Elsevier [103].

Building on this, researchers began deriving metal oxide nanostructures from MOFs to further improve conductivity and sensing performance. For instance, ZnO, a widely studied metal oxide semiconductor, has been extensively explored as a gas-sensing material. In this work, porous ZnO nanocubes derived from MOFs were developed to construct an ultra-highly sensitive and selective chemiresistive NO_2_ sensor [103]. Figure 7, preparation of ZIF-8 and its pyrolyzed derivatives. ZIF-8 was synthesized from Zn(NO_3_)_2_·6H_2_O and DMF in ethanol, aged, washed, and vacuum-dried. The precursor was then pyrolyzed in air at 300–600 °C to obtain products denoted as ZIF-8–300, ZIF-8–350, ZIF-8–400, ZIF-8–450, ZIF-8–500, ZIF-8–550, and ZIF-8–600. The MOF precursors were transformed into ZnO nanocubes through carefully controlled pyrolysis, with the 500 °C treatment yielding the highest sensitivity of 51.41 toward 1 ppm NO_2_ at 200 °C. The sensor also exhibited excellent selectivity over other interfering gases, including CO, C_2_H_5_OH, H_2_, H_2_S, NO, and NH_3_. The remarkable chemiresistive performance is attributed to the unique MOF-derived nanostructure, which provides abundant exposed active sites and interconnected pore channels, facilitating gas adsorption and efficient modulation of electrical resistance.

Similarly, SnO_2_ nanoparticles derived from Sn-MOFs exhibited outstanding NO_2_ responses (up to 3984.98 at 100 ppm, 200 °C), attributed to oxygen vacancies and heterojunction formation [104]. Gas sensing tests revealed very high responses of 240.60 and 3984.98 toward 10 ppm and 100 ppm NO_2_, respectively, at 200 °C, and a notable response of 47.11 toward 100 ppm NO_2_ even at room temperature (25 °C). The enhanced chemiresistive performance was attributed to the high surface area, abundant oxygen vacancies, formation of SnO_2_–SnO_2_ homojunctions, and SnO/SnO_2_ heterojunctions, which facilitate gas adsorption and efficient modulation of electrical resistance. This MOF-derived synthesis method demonstrates a versatile route for producing high surface area metal oxide nanoparticles for advanced gas sensor applications.

Further optimization of Sn-based MOF derivatives led to sensors such as SnO_2_-M-OV-300, which achieved remarkable responses of 11,677 at 120 °C and strong activity even at 50 °C, enabled by oxygen vacancies and active Sn sites [105]. These studies underscore the power of MOF-derived oxides in advancing chemiresistive sensor technology. Density functional theory (DFT) analysis revealed that oxygen vacancies and Sn atoms serve as key active sites, with oxygen vacancies playing the dominant role in NO_2_ adsorption and charge transfer. This synergy between structural defects and MOF-derived architecture enables outstanding chemiresistive performance, highlighting the promise of SnO_2_-M-OV-T sensors for reliable, real-time NO_2_ monitoring in industrial applications.

Expanding beyond Sn and Zn systems, other metal-based MOFs have also been explored. Ti-MOFs synthesized from terephthalic or pyromellitic acid linkers showed strong NO_2_ sensitivity at room temperature, with Ti-MOF(PMA) outperforming its terephthalate counterpart [106]. Among them, Ti-MOF(PMA) showed superior chemiresistive performance, with dynamic responses of 6.4, 16.1, 35.6, and 48.5 to NO_2_ at 50–200 ppm under room temperature. The Ti-MOF(PMA) sensor also exhibited excellent reproducibility and selectivity toward NO_2_ compared with acetone, methanol, ethanol, ammonia, and CO_2_.

Meanwhile, MOF-derived ZnFe_2_O_4_ microparticles integrated with reduced graphene oxide enabled the fabrication of stretchable NO_2_ sensors with excellent performance under mechanical stress and humidity, suitable for e-skin devices [107]. The mesoporous ZnFe_2_O_4_, rich in defect sites, enhanced the rGO sensor’s sensitivity and response to NO_2_, achieving fast detection over a wide range (50–4000 ppb), high sensitivity (219.44% ppm^−1^), and an ultralow detection limit (1.49 × 10^−4^ ppm). The composite sensor maintained stable, reproducible performance under stretching, high humidity, and low-concentration conditions, highlighting its strong potential for wearable gas sensing technologies.

Another approach focuses on leveraging intrinsic MOF properties without full conversion to oxides. Small et al. reported chemically stable, low-power sensors are essential for direct electrical detection of toxic gases, and MOFs provide the structural tunability needed for this purpose [108]. This study investigated how coadsorbed gases affect trace NO_2_ detection in Ni-MOF-74, combining synchrotron diffraction and pair distribution function analyses with electrical measurements. Sixteen gas mixtures (N_2_, NO_2_, SO_2_, CO_2_, and H_2_O at 50 °C) were examined. Results revealed that NO_2_ binding within Ni-MOF-74 pores caused significant resistance drops (up to 6 × 10^3^), strongly amplified by the presence of competitive gases except CO_2_. In contrast, H_2_O alone induced minor, rapid resistance decreases, distinguishable from the slower NO_2_ response via capacitance analysis. Structural studies showed that H_2_O also expanded the lattice and increased charge delocalization. Impedance analysis indicated two distinct electrical processes during NO_2_ adsorption, with the faster one inhibited by CO_2_. Overall, the findings highlight that selective NO_2_ interactions with Ni-MOF-74 dominate the chemiresistive sensing response, while coadsorbed gases (H_2_O, SO_2_, CO_2_) modulate the sensitivity and selectivity. This understanding offers pathways to optimize MOF-based gas sensors for real-world applications.

Likewise, MOF-derived cobalt oxide nanosheets (Co_3_O_4_ NS) exhibited high sensitivity and stability for H_2_S detection, confirming the versatility of MOF precursors in designing high-performance sensors [109]. The Co_3_O_4_-based sensor exhibited excellent chemiresistive performance, with high sensitivity across 0.5–100 ppm H_2_S, achieving a maximum response of 1702.61% at 250 °C for 100 ppm. It further demonstrated a low detection limit of 500 ppb, fast response/recovery times (63.56/103.34 s), and reliable stability over 30 days. These results highlight the potential of MOF-derived nanostructures in developing high-performance H_2_S sensors for practical environmental applications.

To enhance selectivity, researchers have engineered hybrid architectures that combine molecular sieving with catalytic activity. A Co-doped ZnO/ZIF hybrid, for instance, delivered a 130-fold improvement in H_2_S selectivity by tuning oxygen adsorption and base site density [110]. The introduction of Co increased the surface area, promoted oxygen species adsorption on the ZIF surface, and facilitated catalytic oxidation of H_2_S. The optimized sensor demonstrated a strong response of 260 at 5 ppm H_2_S, a low detection limit of 70 ppb, and excellent performance at 180 °C. Beyond molecular sieving effects that block larger interfering gases, Co doping also tuned the base site density in the ZIF layer, minimizing interference from smaller basic molecules. As a result, the device achieved a 130-fold improvement in H_2_S selectivity against various interfering gases and a 54-fold enhancement compared to sensors without Co. These advances establish the sensor as an effective platform for detecting trace H_2_S in applications such as pesticide residue monitoring and food quality assessment.

Similarly, in situ grown Co-MOF array films annealed into Co_3_O_4_ sensors achieved rapid triethylamine detection due to abundant oxygen vacancies and well-aligned conducting channels [111]. The precursor films were subsequently annealed to form in situ Co_3_O_4_ sensors. Morphology, structure, contact properties, and oxygen species of the films were systematically analyzed, revealing their critical influence on triethylamine sensing. The optimized sensor displayed outstanding performance, including a fast response time (reduced from 82 s to 9 s), high sensitivity (R_g_/R_a_ = 230), and improved selectivity relative to Co_3_O_4_ sensors derived from non-MOF precursors. The enhanced triethylamine sensing was attributed to abundant oxygen vacancies and effective conducting channels. This approach offers a simple and scalable strategy for fabricating high-performance in situ gas sensors.

Beyond binary composites, MOFs with tailored pore structures and functional groups have demonstrated superior sensing properties. Ultramicroporous Co-based frameworks such as {[Co_6_(5-MIA)_5_(tmbpy)(μ_3_-OH)_2_]}_n_ selectively adsorbed CO_2_ and C_2_H_2_, achieving high IAST selectivities and excellent reusability [112]. Featuring 3.5 Å apertures and multifunctional sites (open Co^2+^ and methoxy groups), enables synergistic size-sieving and host–guest interactions, facilitating selective adsorption of CO_2_ and C_2_H_2_. Ideal Adsorbed Solution Theory (IAST) selectivities for C_2_H_2_/CH_4_ (1:1) and CO_2_/CH_4_ (1:1) were 26.6 and 12.3, respectively, at ambient conditions.

Likewise, chrysanthemum-like Ni-MOFs (Ni-VNU-74) exhibited strong CO responses, with pore size and exposed Ni^2+^ sites governing sensitivity. Nguyen et al. reported a two iso-reticular Ni-based MOFs, Ni-VNU-74-I and Ni-VNU-74-II, were investigated for CO gas sensing [113]. The materials exhibited chrysanthemum-like morphologies, high crystallinity, excellent thermal stability, and large surface areas (up to 2350 m^2^/g), with distinct pore sizes. Gas-sensing tests revealed that the Ni-VNU-74-II sensor showed a stronger response (R_a_/R_g_ = 1.7) to 50 ppm CO at 200 °C compared to Ni-VNU-74-I (R_a_/R_g_ = 1.2), attributed to enhanced interactions between the CO molecules’ quadrupole moment and the partially charged open Ni^2+^ metal sites. The superior performance of Ni-VNU-74-II was linked to its smaller pores, higher surface area, and increased concentration of exposed Ni^2+^ sites, which promote stronger host–guest interactions and efficient adsorption. These results demonstrate the promise of Ni-MOFs as effective materials for CO detection in practical applications.

Meanwhile, advances in capacitive and QCM-based MOF sensors have expanded resistive sensing into broader transduction platforms. For example, Mg-MOF-74 integrated into QCM devices achieved room-temperature CO_2_ detection with high linearity and stability [114]. The CO_2_ sensing performance of the M-MOF-74-coated QCM sensors was systematically evaluated. Notably, the Mg-MOF-74 sensor exhibited a high response to 2000 ppm CO_2_, with excellent linearity, rapid response/recovery times (75 s/50 s), and good selectivity. Impedance analysis confirmed stable electrical parameters and QCM quality factor. The CO_2_ adsorption mechanism is primarily attributed to the open metal sites in Mg-MOF-74 and the synergistic interaction of Mg–O bonds. These findings highlight M-MOF-74 as a promising material for room-temperature QCM-based CO_2_ sensing applications.

Similarly, Ni-MOF composites with hydroxyl-functionalized CNTs enhanced SO_2_ sensing performance by improving conductivity and adsorption affinity [115]. The sensing mechanism relied on monitoring variations in electrical resistance upon exposure to SO_2_ at room temperature. Compared to pristine Ni-MOF, the composites exhibited superior selectivity toward SO_2_ over NO_2_, NH_3_, and CO within the 0.5–15 ppm range. Among them, Ni-MOF/–OH-SWNTs demonstrated the highest sensitivity (0.9784), rapid response time (10 s), and fast recovery (30 s) at 1 ppm SO_2_, highlighting the synergistic effect of CNT functionalization in boosting MOF-based gas detection performance.

Recent efforts have also emphasized selectivity and stability for toxic gases such as SO_2_. Ni_2_(dobpdc) [116]. The material’s gas sensing method is based on fluorescence, where it shows remarkable selectivity for detecting SO_2_ over other gases. Furthermore, through time-resolved photoluminescence experiments, the study proposes a plausible mechanism for how this nickel-based MOF material selectively detects SO_2_ molecules. Moreover, Zhai et al. reported the MOF Ni_2_(dobpdc) demonstrates exceptional properties for sulfur dioxide (SO_2_) interaction, exhibiting high chemical stability, a significant adsorption capacity of 4.3 mmol g^−1^ at low pressures, and excellent cycling performance for repeated capture and release [117]. The material’s gas sensing method is based on fluorescence, where it shows remarkable selectivity for detecting SO_2_ over other gases. Furthermore, through time-resolved photoluminescence experiments, the study proposes a plausible mechanism for how this nickel-based MOF material selectively detects SO_2_ molecules.

Brandt et al. conducted a comparative analysis of various porous materials for SO_2_ adsorption, evaluating Metal–Organic Frameworks (MOFs) like NH_2_-MIL-101(Cr), HKUST-1, and Basolite F300 and zeolitic imidazolate frameworks (ZIFs) like ZIF-8 and ZIF-67, as well as Zeolite Y, SAPO-34, silica gel, a covalent triazine framework (CTF-1), and active carbon [118]. The gas sensing methodology involved measuring low-pressure SO_2_ uptake capacity and SO_2_/CO_2_ selectivity under dry and humid conditions at 293 K and 1 bar. Key performance results indicate that microporous materials with pores between 4–8 Å or containing nitrogen heterocycles are optimal for SO_2_ uptake. While uptake capacity at 1 bar generally correlates with surface area and pore volume, selectivity is highly material-dependent. Zeolite Y and SAPO-34 were identified as top performers due to their stability in humid environments and exceptional selectivity. Zeolite Y achieved the highest ideal adsorbed solution theory (IAST) selectivity for SO_2_/CO_2_ (265-149), while CTF-1 also showed strong results (63-43), making them the most promising adsorbent materials for flue gas separation.

Moreover, Zhou et al. developed a sensor material by synthesizing ZnFe_2_O_4_ nanorods from a MOF precursor and compositing them with reduced graphene oxide (rGO) via a hydrothermal method to form a ZnFe_2_O_4_/rGO nanocomposite [119]. The gas sensing methodology involved testing the film sensors against SO_2_ concentrations ranging from 1 to 100 ppm at room temperature. The performance of the composite was directly compared to sensors made from the individual components (ZnFe_2_O_4_ and rGO alone). The ZnFe_2_O_4_/rGO composite demonstrated superior sensing performance, exhibiting a high response of 18.32% to SO_2_ at room temperature. It also showed better sensitivity, selectivity, and a faster transient response than its constituent materials. This enhancement is attributed to the formation of a p-n heterostructure between the ZnFe_2_O_4_ nanorods and rGO nanosheets, combined with the excellent electrical conductivity of the rGO itself, providing a new strategy for room-temperature SO_2_ detection.

**Figure 8:**
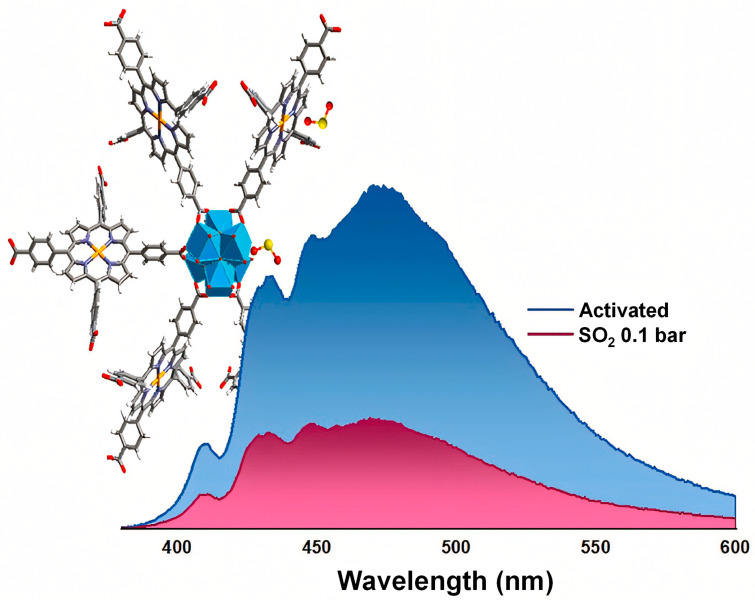
Interaction of SO_2_ molecules with defect-engineered (Hf)PCN-224(Co) featuring missing-linker sites, and the corresponding fluorescence emission spectra of the activated framework before and after exposure to 0.1 bar SO_2_. Reproduced with permission from Elsevier [120].

Additionally, studies show the first experimental and theoretical investigation of a porphyrin-based metal–organic framework (PMOF), (Hf)PCN-224(Co), for SO_2_ sensing [120]. As illustrated in Figure 8, the material exhibits crystallographic defects in the form of missing linkers, creating open Hf(IV) metal sites that facilitate strong coordination with SO_2_ molecules. The framework functions as a photoluminescent sensor, showing a distinct emission response to SO_2_ compared to CO_2_ and water vapor, with a moderate adsorption capacity and a detection limit of 175.5 ppm. The sensing mechanism involves ligand-to-metal charge transfer induced by SO_2_ binding, as confirmed through spectroscopic analysis and density functional theory (DFT) calculations.

For example, a novel porous Zr(IV)-based MOF (HBU-20) was designed and synthesized as an efficient sensor material for toxic gas detection, particularly SO_2_. HBU-20 exhibits a high BET surface area (1551.1 m^2^/g) and a large pore volume (0.896 cm^3^/g), enabling enhanced gas adsorption [121]. The material demonstrates an exceptional SO_2_ uptake capacity of 6.69 mmol/g at 298 K and 100 kPa, along with remarkable IAST selectivities for SO_2_ over CO_2_ (56.7) and CH_4_ (246). Theoretical simulations reveal that strong host–guest interactions between SO_2_ molecules and the MOF are key to its superior sensing and capture performance. Dynamic breakthrough experiments further confirm its high separation efficiency, with experimental selectivities of 81.1 (SO_2_/CO_2_) and 117.6 (SO_2_/CH_4_). These results highlight HBU-20 as a promising MOF-based material for sensitive and selective toxic gas detection and deep desulfurization applications.

Likewise, MIL-53(Al)-TDC and MIL-53(Al)-BDC were investigated for their potential in SO_2_ sensing and adsorption. MIL-53(Al)-TDC exhibits a rigid framework behavior during SO_2_ adsorption, while MIL-53(Al)-BDC demonstrates guest-induced flexibility, as revealed by molecular simulations showing three distinct pore-opening phases: narrow pore, intermediate pore, and large pore [122]. Both materials display high SO_2_ adsorption capacities, excellent stability under wet conditions, good recyclability, and easy regeneration. Owing to these characteristics, MIL-53(Al)-TDC emerges as a promising candidate for SO_2_ sensing applications, while MIL-53(Al)-BDC shows strong potential for SO_2_ storage and transportation.

Zhai et al. reported a Zr-based MOFs have shown significant promise for gas detection, but their powder form limits device integration due to poor air-permeability and flexibility [123]. To overcome this, a green strategy was employed to fabricate a flexible sensing layer by incorporating UiO-66-NH_2_ into nanofibers via electrospinning and aqueous synthesis. The resulting capacitive sensor, composed of the UiO-66-NH_2_ nanofiber membrane and CNTs, leverages high porosity, well-dispersed adsorption sites, and mechanical flexibility to achieve excellent sensitivity and long-term stability for SO_2_ detection from 125 ppm down to 1 ppm. The sensor maintains almost unchanged response over one month and demonstrates a highly linear response (R^2^ = 0.996) at low concentrations. Additionally, it retains 73.33% of its sensing performance even after 24 h of washing and exhibits superior selectivity toward SO_2_ compared to other hazardous gases, making it a robust platform for flexible toxic gas sensing.

**Figure 9:**
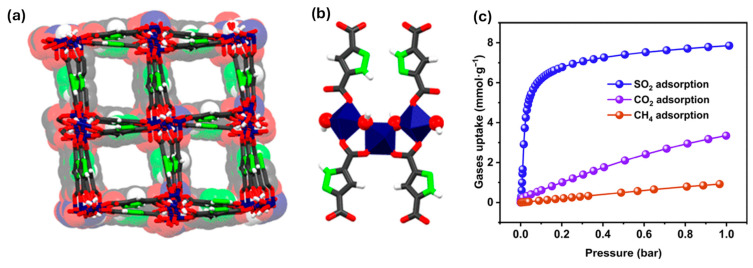
(**a**) Structure of MOF-303: view along the channel c-axis and (**b**) metal cluster and linker arrangement along the b-axis. Atoms are labeled as follows: blue, AlO_4_(μ-OH)_2_ octahedra; gray, carbon; white, hydrogen; green, nitrogen; red, oxygen. (**c**) Single-component adsorption isotherms of SO_2_ (blue), CO_2_ (purple), and CH_4_ (red) at 298 K and 1 bar. Reproduced with permission from ACS [124].

In another study, Obeso et al. highlighted that catalysts for CH_4_ oxidation are frequently poisoned by sulfur-containing gases such as H_2_S and SO_2_, presenting major challenges for practical energy applications [124]. To mitigate this issue, materials capable of selectively capturing SO_2_ are highly desirable. In this context, the water-stable MOF-303 exhibits high SO_2_ adsorption at low pressures (6.21 mmol·g^−1^ at 298 K and 0.1 bar), making it well-suited for detecting and removing trace SO_2_. Figure 9 illustrates the structural features and adsorption properties of MOF-303, which underlie its selective gas uptake. The material also exhibits excellent chemical stability under both dry and humid conditions, outstanding cycling performance, and easy regeneration. Moreover, MOF-303 shows high selectivity for SO_2_ over CH_4_ and CO_2_. Fluorescence experiments further confirm its promising SO_2_ sensing capability, even in the presence of competing gases, highlighting its potential as an effective material for toxic gas detection and protection of CH_4_ oxidation catalysts.

The detection of corrosive gases such as SO_2_ remains highly challenging due to their aggressive nature. To address this, a bio-derived amino-functionalized MOF, Bio-ZJU-928(Ni), was developed featuring a nickel paddle-wheel secondary building unit, acetic acid ligand, and adenine with amino groups [125]. This material demonstrated high chemical and thermal stability, a surface area of 634 m^2^/g, and remarkable trace SO_2_ uptake (2.94 mmol/g) at 0.002 bar and 273 K. It also exhibited excellent selectivity with high IAST values for SO_2_/N_2_ (32,145) and SO_2_/CO_2_ (259.35) at 10/90 (*v*/*v*), while maintaining strong adsorption under humid conditions. Enhanced performance was attributed to hydrogen-bond formation with water molecules, as confirmed by in situ DRIFTS and Monte Carlo simulations, suggesting strong potential for efficient SO_2_ capture and sensing.

Moving to VOCs, a significant challenge lies in distinguishing structurally similar isomers in mixtures. To tackle this, a MOF-based electronic nose (MOF-e-nose) was developed using an array of six QCM sensors coated with selected MOF films [126]. MOFs, with their well-defined nanoporous structures and tunable adsorption properties, offer a promising route to enhance VOC sensing. In this study, ternary xylene isomer mixtures were detected and identified using an array of six QCM sensors coated with selected MOF films, each tailored for different isomer affinities. Classical molecular simulations provided insight into the sensing mechanism, revealing that isomer discrimination arises not only from strong analyte–MOF interactions but also from the rigid crystalline frameworks sterically controlling access to specific adsorption sites. The sensor array demonstrated a low detection limit of 1 ppm for individual isomers and enabled accurate discrimination in mixtures. At 100 ppm, 16 different ternary o–p–m-xylene mixtures were identified with 96.5% classification accuracy. These results highlight the exceptional performance of MOF-based sensor arrays, or MOF-electronic noses (MOF-e-noses), and provide valuable guidelines for detecting and distinguishing complex VOC mixtures.

Beyond electronic noses, MOF composites have also been applied to resistive sensing. A MIL-101(Cr)/CNT nanocomposite was reported for the first time as a room-temperature resistive VOC sensor [127]. Leveraging the high porosity of MIL-101(Cr) and the conductivity of CNTs, the composite successfully detected multiple VOCs (e.g., methanol, ethanol, acetone, dichloromethane) with good sensitivity, repeatability, and stability. This highlights the potential of MOF–carbon composites in overcoming MOFs’ inherent dielectric nature, enabling their integration into practical resistive devices.

In another approach, MOF-derived oxides were applied for VOC detection. One-dimensional SnO_2_@NiO core–shell nanofibers were synthesized via a solvothermal process followed by heat treatment, with morphology tuned by reaction time [128]. The optimized sensor showed high selectivity and sensitivity to xylene (R_a_/R_g_ = 25.6), while other variants preferentially detected acetone (R_a_/R_g_ = 28.7) and triethylamine (R_a_/R_g_ = 13.7). Structural studies confirmed a p–n heterojunction with abundant oxygen vacancies and Ni^3+^ sites, which facilitated charge transfer and gas adsorption. In situ FTIR and spectroscopic analyses revealed surface intermediates and catalytic redox processes as the key mechanisms, underscoring the advantages of MOF-derived strategies for high-performance VOC sensing.

Sustainable substrates have also emerged as alternatives for MOF growth. A robust method was developed to grow MOFs on fibrous banana paper (BP) via vapor-phase synthesis, utilizing lignocellulosic biomass as the substrate [129]. The resulting BP-MOF composites exhibit strong antibacterial activity (99.2% *E. coli* elimination within 1 h) and show enhanced absorption of 1-octen-3-ol vapor in preliminary smartphone-based VOC sensing studies, highlighting their potential for VOC capture and detection. This sustainable, flexible, and hierarchically porous MOF-fiber composite offers a promising platform for antibacterial applications and environmental monitoring, utilizing waste banana biomass as a cost-effective and versatile substrate.

Photonic MOF sensors provide another avenue for VOC detection. Inspired by beetle cuticle structures, a porous photonic crystal (PC) sensor was fabricated by combining ZIF-8 with TiO_2_@PDA nanoparticles [130]. The device exhibited distinct color changes in response to benzene, with a detection limit of 0.8 g/m^3^, response times under 1 s, and stable performance over 100 cycles. Comparative studies showed that ZIF-8 outperformed ZIF-67 and ZIF-7 in benzene sensing. QCM-D analysis confirmed synergistic adsorption within the MOF layers, providing insights into how microscopic adsorption events translate to macroscopic optical signals.

Finally, optical integration of MOFs with silicon photonics was reported by Ma et al., who assembled ZIF-8 nanomaterials onto SiO_2_ waveguides to create an evanescent wave sensing platform [131]. The functionalized waveguides were incorporated into asymmetric Mach–Zehnder interferometers (AMZIs), achieving an ultra-high extinction ratio (28.6 dB), low insertion loss (~13 dB), and broad spectral response. The sensor demonstrated excellent ethanol detection performance, with a wide detection range (0–1000 ppm), high sensitivity (19 pm ppm^−1^ for 0–50 ppm, 41 pm ppm^−1^ for 600–1000 ppm), and low detection limits (1.6 ppm or 740 ppb). This integration of ZIF-8 nanomaterials with optical waveguides highlights a promising route for lab-on-waveguide VOC sensing platforms with high sensitivity and broad applicability.

Taken together, these studies reveal distinct advantages and limitations across different MOF-based sensing strategies. Bio-derived frameworks such as Bio-ZJU-928(Ni) excel in SO_2_ adsorption and stability but remain highly system-specific. MOF-e-noses enable powerful VOC discrimination but require complex sensor arrays and simulations for calibration. Hybrid composites such as MIL-101(Cr)/CNT achieve room-temperature resistive sensing with high stability, yet scalability of nanocomposite fabrication may be limiting. MOF-derived oxides like SnO_2_@NiO nanofibers offer high sensitivity and tunable selectivity through heterojunction effects, though they often demand energy-intensive synthesis. Sustainable BP-MOF substrates promote eco-friendly and flexible sensing but are still at the proof-of-concept stage. Photonic and waveguide-integrated MOF sensors provide rapid, visual, and highly sensitive detection, but fabrication complexity and integration costs could hinder near-term commercialization. Thus, the central trade-off lies between sensitivity vs. practicality, selectivity vs. scalability, and sustainability vs. integration complexity, highlighting the need for balanced material design strategies to achieve reliable, real-world environmental sensing solutions.

#### 4.2.2. Optical (Fluorescence, Luminescence, Colorimetric)

MOFs with photoactive linkers or luminescent metal centers have emerged as versatile platforms for gas detection. Their optical signals, fluorescence quenching, emission shifts, or even simple color changes can be highly sensitive to analytes and are appealing for non-invasive, real-time monitoring of toxic gases [132]. A colorimetric sensor array was designed to enable rapid and sensitive detection of 20 toxic industrial gases at their permissible exposure limits (PELs) [133]. The system utilizes nanoporous, chemically responsive pigments that exhibit distinct color changes upon gas exposure, allowing straightforward visual identification of each analyte with an error rate of less than 0.7%. Still, the degree to which these signals remain stable in practical environments varies from system to system.

One illustrative example comes from Fan et al., who synthesized a Eu-based framework, {[Eu_2_(L)(phen)_2_(ox)_2_(H_2_O)_2_]·10H_2_O·phen}_n_ (H_2_L = 2,6-bis(4-carboxyphenyl)pyrazine, phen = 1,10-phenanthroline), and showed that it could act as a multifunctional luminescent sensor for benzaldehyde (gas/liquid), Hg^2+^, and Cr_2_O_7_^2−^/CrO_4_^2−^ [134]. The material displayed high selectivity and quenching-based sensitivity, while remaining stable across a broad pH range. Perhaps more importantly, it was tested in realistic settings, tap water, tea, and river water, where recovery rates approached 100%. The group even demonstrated portable fluorescent strips for benzaldehyde detection, which points to actual field applicability rather than just a laboratory proof-of-concept.

Other studies have pushed toward gas-specific frameworks. MUF-16, a Co(II)-MOF with amino-functionalized linkers, exhibited reversible SO_2_ uptake (2.2 mmol g^−1^ at 1 bar) and selective fluorescence quenching [135]. The framework discriminated well against CO_2_, NO_2_, CH_4_, and other common gases, achieving a detection limit of 80.72 ppm. While promising, this level of selectivity is often obtained under controlled conditions; long-term operation in humid or industrial environments may not be as straightforward.

Molecular probes have also carved out space in this landscape. Jing et al. introduced Mito-Na-BP, a mitochondria-targeted two-photon probe for simultaneous SO_2_ and glutathione (GSH) detection [136]. Under single-wavelength excitation, the sensor displayed distinct fluorescence responses: blue-shifted signals for GSH-to-SO_2_ conversion and green-enhanced signals for SO_2_-to-GSH conversion, while individual GSH and SO_2_ detection showed opposite fluorescence trends at 638 nm. With high sensitivity, selectivity, and rapid response at physiological pH, Mito-Na-BP was successfully applied for real-time monitoring of GSH fluctuations induced by SO_2_ and visualizing GSH-to-SO_2_ metabolic processes via TP imaging. This multifunctional fluorescence sensor provides a convenient tool for exploring the dynamic relationship between SO_2_ and GSH and offers potential for biomedical studies on their physiological roles.

Another probe, HBT-EMBI, used ESIPT and ICT mechanisms to track SO_2_ derivatives in food and live cells with sharp ratiometric signals [137]. The probe exhibits distinct colorimetric changes (purple to colorless), ratiometric fluorescence with zero cross-talk, and a large emission shift (Δλ = 164 nm) under single-wavelength excitation. HBT-EMBI has been successfully applied for colorimetric and ratiometric detection of SO_2_ derivatives in real food samples and for quantitative visualization of SO_2_ variations in HepG2 cells, demonstrating its potential for practical food safety monitoring and cellular studies.

Framework design strategies continue to refine SO_2_ sensing. The Al(III)-based MOF CYCU-3 demonstrates significant SO_2_ adsorption, achieving 11.03 mmol g^−1^ at 1 bar and 298 K, and exhibits high chemical stability under both dry and wet SO_2_ conditions [138]. DRIFTS measurements and computational studies identified hydrogen bonding with bridging Al(III)–OH groups as the primary adsorption sites. For SO_2_ sensing, photoluminescence experiments revealed a selective turn-on fluorescence response over CO_2_ and H_2_O. The sensing mechanism is attributed to suppression of ligand–metal energy transfer and enhancement of ligand-centered π → π transitions*, highlighting CYCU-3’s potential as a fluorescent MOF sensor for selective SO_2_ detection.

Beyond MOFs, covalent organic frameworks (COFs) have emerged as strong contenders. An imine-functionalized COF, SonoCOF-9, exhibited reversible SO_2_ adsorption of 3.5 mmol g^−1^ at 1 bar and 298 K, and 0.91 mmol g^−1^ at 0.1 bar, maintaining excellent reversibility over 50 adsorption–desorption cycles [139]. The isosteric heat of adsorption (ΔH_a_d_s_ = −42.3 kJ mol^−1^) indicates strong SO_2_ COF interactions, which were further confirmed by molecular dynamics simulations and Møller–Plesset perturbation theory calculations, showing SO_2_ binding to the π-electron density of the rings and N lone pairs. These results demonstrate the potential of SonoCOF-9 as a selective SO_2_ sensor, capable of detecting SO_2_ at the sub-ppm level (0.0064 ppm), combining both experimental and theoretical validation for its sensing performance.

Hybrid approaches expand the picture further. Che et al. reported that a novel gas sensor was developed by covalently linking a coumarin fluorophore to an anion-functionalized ionic liquid (IL) for real-time, visual detection of gaseous SO_2_ [140]. The sensor operates via fluorescence signaling induced by strong chemical interactions with SO_2_ and exhibits high sensitivity and selectivity, detecting trace levels below 0.2 ppm. The IL-based sensor overcomes water sensitivity and interference issues and, due to its excellent solubility, can be fabricated into a simple, portable membrane device with reusability over 12 cycles. This approach provides a next-generation SO_2_ gas sensor with superior performance, suitable for environmental and industrial monitoring of harmful gases.

**Figure 10:**
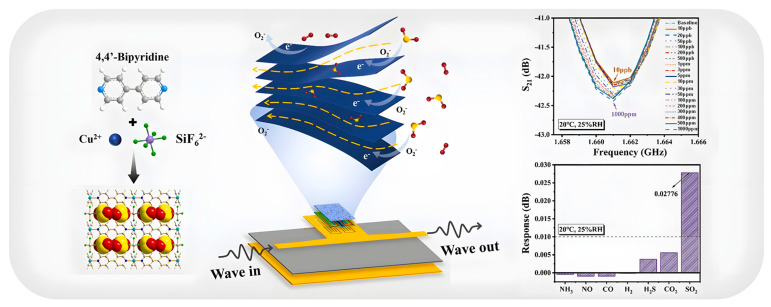
Illustration of the SIFSIX-1-Cu microwave gas sensor for SO_2_ detection, where adsorption in the MOF’s nanochannels induces changes in electromagnetic properties, allowing sensitive, selective, and moisture-tolerant detection at ambient conditions. Reproduced with permission from Elsevier [141].

A different route, the microwave gas sensor (MGS) incorporating SIFSIX-1-Cu, circumvented the conductivity limitations of MOFs entirely. By relying on dielectric constant shifts in nanochannels, the device reached ppb-level SO_2_ detection (LOD = 8.9 ppb) with high moisture tolerance and a broad detection range (10 ppb–1000 ppm) [141]. Figure 10, schematic of the SIFSIX-1-Cu microwave gas sensor (MGS) for selective SO_2_ detection. While effective, such setups require more complex instrumentation than straightforward fluorescence readouts.

Efforts are not limited to sulfur-containing gases. Rh6G@UiO-66-NH_2_ was shown to detect nitrite ions through diazonium salt formation, with a detection limit of 0.021 μM, and even incorporated into portable hydrogel kits for on-site testing [142]. This approach enabled a linear response between NO_2_^−^ concentration and fluorescence intensity ratio across 1–100 μM. The kit allows rapid NO_2_^−^ analysis via smartphone-assisted imaging and supports detection over 0.1–1.5 mM with accurate quantification, demonstrating its practical potential for real-sample monitoring and food safety assessment.

Similarly, a Zn-based luminescent MOF responded sharply to nitro-aromatics like 2,4-DNP and nitrobenzene, achieving 0.284 μM detection limits and recyclability [143]. The fluorescence quenching mechanism was attributed to dipole–dipole interactions and π–π stacking between the MOF and nitro-aromatic molecules. The sensor demonstrated excellent recyclability with >95% fluorescence retention after four cycles, underscoring its high sensitivity, selectivity, and stability for detecting nitro-aromatic explosives and environmental pollutants.

For NO_2_ sensing, Ni–Mg MOF-74 mixed-metal films leveraged impedance detection, where Ni boosted both framework stability and electron density [144]. Density functional theory (DFT) calculations revealed that Ni incorporation improved framework stability, increased electron density near the HOMO, and strengthened NO_2_ adsorption on Mg sites. Impedance measurements showed that Ni-rich NixMg_1−x_-MOF-74 films exhibited a significantly higher response upon exposure to 1 ppm NO_2_. Among various fabrication methods, the MOF-on-MOF grown Ni-on-Mg-MOF-74 films demonstrated the best sensing performance, achieving an impedance change of 309, which is 52% higher than the best-performing monometallic Ni-MOF sensor. Structural analyses confirmed that the Mg-MOF-74 base layer served as a templating scaffold, leading to improved crystallinity, larger grain sizes, and superior film quality, ultimately enhancing sensing efficiency. This work highlights a synergistic material-engineering strategy combining metal mixing and MOF-on-MOF templated growth to design high-performance impedance-based MOF sensors for ultra-sensitive NO_2_ detection.

ZJU-66 films, fabricated by substrate oxidation, provided another strategy for sensitive fluorescence quenching [145]. The detection mechanism is attributed to the suppression of excitation and emission processes caused by the strong electron-absorbing capacity of NO_2_ molecules, resulting in highly sensitive fluorescence quenching. This strategy provides a general approach for producing high-quality, stable MOF films with potential applications in toxic gas detection and other sensing platforms.

In biomedical and environmental contexts, nitric oxide (NO) sensing has also been explored. PABA@MOF-808, obtained through post-synthetic modification, exhibited selective fluorescence quenching by deamination, with a detection limit near 21 ppb [146]. This functionalized MOF enabled selective and sensitive detection of NO in both aqueous and gaseous environments. The sensing mechanism follows a fluorogenic “turn-off” response, where NO induces fluorescence quenching through a deamination reaction, exhibiting excellent selectivity over other reactive nitrogen and oxygen species (RNS and ROS). The sensor demonstrated a high Stern–Volmer constant (Ksv = 6.10 × 10^3^ M^−1^) and an exceptionally low LOD, highlighting its superior sensitivity. For practical applications, a flexible mixed-matrix membrane (MMM) of PABA@MOF-808 was developed, enabling efficient NO sensing in both water-based systems and vapor phase detection. Experimental findings combined with theoretical insights further elucidated the underlying sensing mechanism.

Notably, this MOF functioned in both aqueous and gaseous environments using flexible membranes, hinting at practical versatility. Beyond single-phase systems, heterojunctions have further advanced detection. A CoNiHHTP MOF/PHI Z-scheme heterojunction achieved ultra-sensitive NO_2_ detection (LOD = 1 ppb), rapid response/recovery, and stability over 150 days [147]. The optimized heterojunction achieved a record NO_2_ detection limit of 1 ppb (response = 15.6%) under 405 nm irradiation at RT, with rapid response (3.6 min) and recovery (2.7 min) times, high selectivity and reversibility, and long-term stability up to 150 days, outperforming pristine PHI and most reported sensors at RT. Notably, the bimetallic Co–Ni configuration mitigates interference from O_2_, further improving NO_2_ sensing performance. This study demonstrates a feasible strategy for designing PHI-based optoelectronic gas sensors with ultrasensitive, selective, and stable NO_2_ detection at room temperature.

Integration with digital platforms is also gaining traction. For instance, Ru@MOF-NH_2_ combined ratiometric fluorescence with smartphone-based readouts for NO_2_^−^, offering naked-eye colorimetric changes alongside ultra-sensitive quantification (LOD = 0.6 μM) [148]. The amino groups of UiO-66-NH_2_ react selectively with NO_2_^−^ through a diazo reaction, producing a blue emission as the reporting signal, while the red emission of [Ru(bpy)_3_]^2+^ serves as a reference. This dual-emission system enables ratiometric detection, allowing a naked-eye color change from blue to red in response to NO_2_^−^. Validation in spiked samples showed accuracy and repeatability ranging from 105–117% with a coefficient of variation below 4.3%, demonstrating its reliability. This POCT ratiometric sensing platform offers a practical strategy for on-site monitoring of nitrite in food and environmental safety applications.

Taken together, these examples underscore both the promise and limitations of MOF- and COF-based optical sensing. Lanthanide frameworks, amino-functionalized systems, and high-capacity designs like CYCU-3 demonstrate strong selectivity and stability, though their analyte scope is narrow. Molecular probes and hybrid strategies achieve ratiometric sensitivity and biological compatibility but are not yet easy to integrate into durable devices. Non-conductive approaches such as microwave sensing address MOF conductivity issues but at the cost of more complex instrumentation. Nitrite, nitro-aromatic, and NO sensors push sensitivity into the ppb–μM range with portable formats, but questions of recyclability and cost linger. Cutting-edge heterojunctions achieve record-low detection limits and long-term stability, though their synthesis remains non-trivial. In short, the recurring trade-offs involve sensitivity versus generality, stability versus fabrication complexity, and biological compatibility versus device scalability. Progress, it seems, will depend less on chasing a single “perfect” MOF and more on designing systems that balance material innovation with practical deployment needs.

#### 4.2.3. Electrochemical Detection

One study developed a nitrite sensor using carbon black/copper MOF (CB/Cu-MOF) nanocomposites immobilized on a screen-printed carbon electrode (SPCE) [149]. The idea was fairly straightforward: carbon black brings conductivity, while the Cu-MOF contributes porosity and active sites. Together, they produced a noticeable boost in electrochemical activity and nitrite adsorption. Tests with cyclic voltammetry, impedance spectroscopy, and linear sweep voltammetry all pointed toward a low detection limit, broad linear range, and fast response. In practice, the device worked well in wastewater, with recoveries close to 100%. Still, it is very much a single-analyte sensor, which raises questions about its utility in more complex monitoring scenarios.

To move beyond that limitation, another team developed a flower-like Cu-doped zeolitic imidazolate framework (Zn0.5/Cu0.5/Co-ZIF) as a ratiometric electrochemical probe for detecting both catechol (CC) and acetaminophen (AP) at once [150]. The synthesis was surprisingly simple, just room-temperature stirring, but Cu doping altered the Zn/Co-ZIF morphology into a more open, flower-like structure that improved electron and mass transfer. The copper sites also acted as an internal reference, helping stabilize signals. By measuring two signals rather than one, the sensor handled complex lake water better than typical single-channel approaches. The catch, though, is that this sort of morphology-controlled synthesis, while clever, may not be easy to scale up.

A different angle came from Feng et al. reported a novel electrochemical sensor based on a hydrophilic carbon cloth electrode modified with Ce/Ni/Cu layered double hydroxide (CeNiCu-LDH@CC) for the simultaneous detection of chlorophenol pollutants, 2,4-dichlorophenol (2,4-DCP) and 3-chlorophenol (3-CP) [151]. The sensor material is derived from a Ce/Ni (CeNi-MOF) precursor, doped with Cu(II), and subsequently alkali-etched to form LDH, which increases surface area and provides abundant adsorption sites. Cu(II) doping enhances the electrode’s conductivity, resulting in an electrochemically active surface area of 9.68 cm^2^. The CeNiCu-LDH@CC electrode exhibits excellent sensing performance, achieving linear detection ranges from 1 to 100 μM for both chlorophenols, with low detection limits of 0.197 μM for 2,4-DCP and 0.286 μM for 3-CP. The sensor also demonstrates high stability, selectivity, and reliable recovery in real sample analyses, highlighting its potential for practical, simultaneous detection of these environmentally relevant toxic compounds. But the synthesis process, being multi-step and somewhat delicate, may be a stumbling block for low-cost or large-scale deployment.

Yet another strategy used a self-standing nanocomposite mat combining Cu(BTC) MOFs with carbon nanofibers (Cu(BTC)MOF@CNF) for enzyme-free 4-nitrophenol detection [152]. The hybrid mat provided ample surface area and functional sites, encouraging fast analyte transport at the electrode–electrolyte interface. Results were strong: a wide linear range (5–400 μM), a low detection limit (87.12 nM), high sensitivity, and good long-term stability. The device resisted interference, held up over 30 days, and even matched results from HPLC analyses. That said, it is not obvious how such mats could be integrated into portable or disposable platforms, which are often needed in environmental fieldwork.

Trade-offs across studies. Stepping back, these examples illustrate a recurring theme in MOF-based electrochemical sensors. The CB/Cu-MOF electrode is simple and reproducible but too narrow in scope. The Cu-ZIF probe handles multiple analytes but depends on morphology control that may complicate scaling. The CeNiCu-LDH@CC electrode combines conductivity and large surface area but requires a multi-step preparation that may not be practical outside the lab. And the Cu(BTC)MOF@CNF mat achieves stability and interference resistance, yet it is unclear how to transition such composites into flexible, real-world devices. What emerges is a familiar balancing act: high analytical performance is achievable, but often at the expense of fabrication simplicity and field deployability. Bridging that gap remains one of the bigger challenges for environmental monitoring applications.

### 4.3. MOF Composites and Hybrid Structures

#### 4.3.1. MOF–Carbon Nanomaterial Hybrids

Pairing MOFs with carbon nanomaterials, such as graphene, CNTs, or rGO, has become a common strategy to overcome the weak spots of pristine MOFs. On their own, MOFs usually fall short in conductivity and charge transport, but carbon frameworks provide exactly what’s missing: pathways for electrons and extra robustness. The result is a class of composites that not only preserves MOFs’ porosity and adsorption selectivity but also gains the speed and stability of carbon.

A case in point is the Fe_3_O_4_@Au/MOF–P_2_W_17_V nanocomposite, layered onto glassy carbon electrodes for nitrite sensing [153]. The vanadium-substituted tungsten phosphate (P_2_W_17_V) and Fe_3_O_4_@Au/MOF were subsequently integrated onto glassy carbon electrodes through layer-by-layer self-assembly and electrodeposition to construct a Fe_3_O_4_@Au/MOF–P_2_W_17_V nanocomposite sensor for nitrite detection. Under optimized conditions, the sensor displayed a linear nitrite detection range of 0.01–100 mM, a sensitivity of 11.682 μA·μM^−1^·cm^−2^, and a detection limit of 0.532 μM. Stability tests showed that the current response retained over 95% after 100 cycles and 30 days. The sensor also exhibited high selectivity and successfully quantified nitrite in ham sausage, squash, milk, and brined quail eggs, producing results consistent with the conventional naphthylenediamine hydrochloride method.

In a slightly different approach, Zn-BDC MOFs coupled with rGO created Zn-BDC@rGO composites that could tell apart gases such as NH_3_, CO, and SO_2_ with the help of PCA [154]. For gas sensing, Zn-BDC@rGO was deposited via drop-casting onto copper electrodes (100 µm gap) on a glass substrate using a shadow mask and e-beam evaporation. The sensor was tested in a chemiresistive mode for NH_3_, CO, and SO_2_ detection. Principal component analysis (PCA) demonstrated that the sensor could selectively discriminate between the three gases, exhibiting fast response/recovery times of 60/120 s at 20 ppm. These values are well below the OSHA permissible exposure limits for CO and NH_3_, and close to the PEL for SO_2_, indicating the sensor’s potential for sensitive and selective monitoring of toxic gases.

Not all efforts have targeted general gases; some push into ultra-trace or biomedical sensing. A Cu-hemin MOF/rGO system, for example, brought NO detection down to ppb levels at room temperature [155]. The material revealed a two-dimensional sheet-like morphology, providing abundant active sites for efficient NO adsorption. The Cu-hemin MOF/rGO sensor demonstrated excellent NO sensing performance, including high sensitivity (R_a_/R_g_ = 1.06 at 50 ppb), fast response/recovery times (43 s/367 s at 10 ppm), strong selectivity, and reliable repeatability. Mechanistic studies indicated that the MOF formation modified the hemin dimer structure, generating additional Fe(III)–N4 active sites, while rGO incorporation enhanced electrical conductivity. Furthermore, a mask-type sensor fabricated from this sheet-like composite confirmed its potential as a flexible and wearable device for ultra-low-level exhaled NO detection.

Vanadium-based MOFs paired with porous graphene also pushed the limits for NO_2_ detection, reporting nearly 800% response at 100 ppm and a recovery time under 40 s [156]. Flexible gas sensors were fabricated using interdigital electrodes on CO_2_ laser-patterned polyimide films combined with porous graphene. The materials were characterized for surface morphology, chemical composition, crystallinity, and specific surface area. Sensor performance, including response, response/recovery time (ts/tc), repeatability, selectivity, and stability, was evaluated using an ultraprecision electrometer. The V-MOF120(PTA) sensor showed the best performance with an average response of 800.8% at 100 ppm NO_2_, fast response/recovery times of 230/39.8 s, a detection limit of 1 ppm, and excellent stability and repeatability.

Biomedical extensions are also emerging. Ni-Zn-MOF/GO hybrids modified with ferrocene successfully quantified glutathione in blood and urine at sub-nanomolar detection limits [150]. A Ni-Zn-MOF/GO/ferrocene (FC)-modified CPE was developed for electrochemical detection of GSH. The sensor exhibited dual linear response ranges for GSH: 0.01–90.0 μM and 90.0–800.0 μM, with a detection limit of 0.003 μM. It demonstrated high selectivity, being able to accurately detect GSH in the presence of tryptophan. The sensor was successfully applied to real samples, including human blood, GSH tablets, and urine, highlighting its potential as a reliable, sensitive, and practical platform for clinical GSH detection.

Likewise, Ce-MOF/CNT composites exploited the Ce^3+^/Ce^4+^ redox shuttle for tracking catechol and hydroquinone simultaneously [157]. Leveraging the high electrical conductivity of CNTs, the mixed valence of Ce, and the large surface area of the MOF, an electrochemical sensor was constructed for the simultaneous detection of hydroquinone (HQ) and catechol (CC). Compared to the untreated Ce-MOF/CNT sensor, the post-treated Ce-MOF(Ce^3+^/Ce^4+^)/CNT electrode exhibited well-defined oxidation peaks for HQ and CC. The sensor showed linear response ranges of 10–100 μM for HQ and 5–50 μM for CC, demonstrating improved electrochemical performance.

In another study, ligand-deficient, layered cerium-based MOF (Ce-MOF) nanosheets were grown on CNT substrates for electrochemical sensing applications. The formation of these 2D MOF nanosheets exploits CNTs as a structural backbone and employs controlled low ligand-to-metal ratios, enabling a layered architecture with hierarchical channels that facilitate efficient electron, ion, and mass transport [158]. The resulting CNTs@Ce-MOF hybrid uses Ce as the electrocatalytic center for nitrite detection, exhibiting two linear response ranges: 0.65–3.25 μM (sensitivity: 87.65 ± 0.6 μA·μM^−1^·cm^2^) and 3.25–7000 μM (sensitivity: 0.35 ± 0.03 μA·μM^−1^·cm^2^), with a detection limit of 0.12 μM (S/N = 3). The sensor demonstrates excellent reproducibility, stability, selectivity, and fast response, outperforming previously reported nitrite sensors.

Toxic gas detection hasn’t been ignored either. The methanol exposure poses severe health risks, especially in regions where adulterated alcoholic beverages are prevalent. Traditional detection techniques, such as gas–liquid chromatography or blood gas analysis, are costly and impractical in low-resource settings. To address this, a chemiresistive sensor was developed using an extrusion-printed hybrid composite of NU-1000 MOFs and graphene, leveraging the high porosity and active metal sites of MOFs along with graphene’s excellent conductivity to achieve rapid, ultrasensitive, and selective methanol detection at ultralow concentrations, even in the presence of high ethanol levels [159]. Machine learning and principal component analysis (PCA) were integrated to enhance discrimination between methanol, ethanol, and other interferents. The extrusion printing technique enabled uniform, stable, and reproducible sensor layers on ceramic substrates. The sensor successfully detected methanol vapors at parts-per-billion (ppb) levels against higher ethanol concentrations, demonstrating its potential for breath analysis, medical diagnostics, and industrial or consumer safety monitoring. This study highlights the promise of extrusion-printed MOF–graphene hybrids for scalable, low-cost, and high-performance gas sensing applications.

Elsewhere, Ce-BTC/GO treated by ion irradiation created fresh adsorption sites, making H_2_S sensing feasible in real time [160]. The material’s composite properties enhance its surface activity by creating additional adsorption sites for H_2_S molecules. This modification leads to dramatic conductivity modulation upon gas exposure, enabling high-performance real-time sensing at concentrations below the OSHA-defined maximum residue limit. The sensor demonstrates excellent response and recovery times, high reproducibility, good repeatability, and long-term stability at room temperature. With a sensitivity of 54.7% across a concentration range of 10–100 ppm and a detection limit of 10 ppm, the SHI-irradiated Ce-BTC/GO composite offers a promising platform for efficient toxic gas detection in environmental and industrial applications.

**Figure 11:**
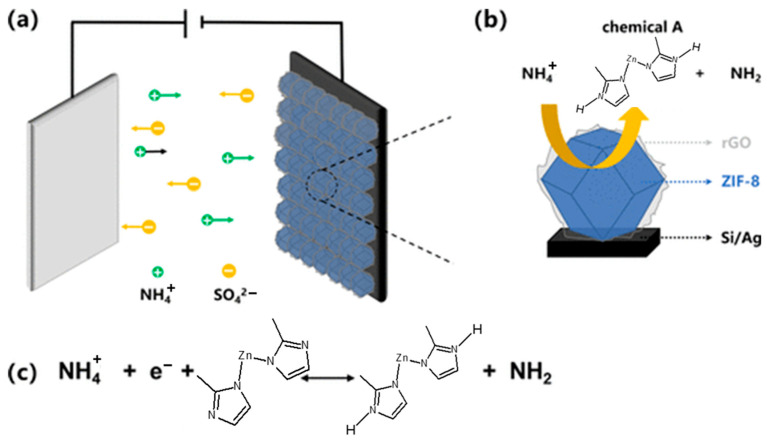
(**a**) Schematic of the DPV-based detection setup. (**b**) Enlarged view of the ZIF-8/rGO working electrode from (**a**). (**c**) Chemical reaction occurring during NH_4_^+^ detection. Reproduced with permission from ACS [161].

And for a very different target, two-dimensional reduced graphene oxide (rGO) and three-dimensional ZIF-8 were used for the detection of ammonium ions (NH_4_^+^) [161]. The ZIF-8/rGO composite is grown in situ and anchored onto a Si/Ag substrate, where the rGO provides high electrical conductivity, and the ZIF-8 contributes a large specific surface area with abundant active sites. Figure 11 illustrates the NH_4_^+^ detection mechanism on the ZIF-8/rGO electrode, showing NH_4_^+^ migration toward the working electrode under −0.05 V, the interaction with ZIF-8/rGO generating NH_2_ groups, and the corresponding DPV measurement setup. This synergistic 2D–3D structure enhances electron transfer, increases conductivity, and improves electrochemical performance. Using differential pulse voltammetry at neutral pH, the composite electrode demonstrates high sensitivity, a wide detection range, and a low detection limit, along with excellent repeatability, stability, and selectivity. The enhanced sensing performance is attributed to the oxidation of NH_4_^+^ facilitated by the ZIF-8 framework and the efficient multidimensional electron transfer channels formed within the composite.

Overall, the gains are clearly better conductivity, sharper selectivity, and broader applicability but they do not come free. Techniques like extrusion printing scale well but need precise deposition control, while irradiation can improve sensitivity at the expense of cost and complexity. Some hybrids may also struggle under high humidity, which could weaken stability. In short, MOF–carbon hybrids are versatile, but their real-world success will depend on balancing performance with manufacturability.

#### 4.3.2. MOF–Polymer Hybrids

If carbon hybrids are about conductivity, polymer hybrids are about flexibility and processability. MOFs embedded in polymer films or membranes inherit the mechanical strength and environmental durability of the host while contributing their own porosity and selectivity. These combinations often make sense for wearable sensors or large-area flexible devices.

A good example is the MOF-5/chitosan membrane, where conductivity was tuned using a glycerol-based ionic liquid [162]. The sensor is fabricated by embedding MOF-5 microparticles into a conductivity-controlled chitosan (CS) organic membrane, where the membrane’s conductivity is finely tuned by incorporating a glycerol-based ionic liquid (IL) at varying concentrations. The resulting MOF-5/CS/IL composite sensor exhibits remarkable sensitivity, enabling the detection of H_2_S at concentrations as low as 1 ppm under ambient conditions. It further demonstrates high selectivity, an ultrafast response time (<8 s), and a short recovery period (<30 s), along with exceptional sensing stability, maintaining 97% detection efficiency at 50 ppm H_2_S. Owing to its high sensitivity, flexibility, low power consumption, and excellent stability, this MOF-based MMM offers significant potential for toxic gas monitoring and addressing key challenges in environmental sustainability.

In another study, Ali et al. reported the development of a flexible MMM sensor based on Cu_3_(HHTP)_2_ metal–organic framework (Cu-MOF) embedded within a polyvinyl alcohol (PVA)/ionic liquid (IL) polymer matrix for the room-temperature detection of H_2_S [163]. The membrane, fabricated by casting a homogeneous suspension of Cu-MOF particles, PVA, and IL onto a Petri dish, resulted in a thin, flexible membrane (215 μm). Structural and morphological analyses confirmed the successful integration of Cu-MOF within the polymer matrix. The resulting Cu-MOF/PVA/IL sensor demonstrated high sensitivity, achieving a detection limit of 1 ppm H_2_S at 23 °C, along with a rapid response time of 12 s. The sensor also exhibited excellent repeatability, long-term stability, and strong selectivity toward H_2_S over other gases. Owing to its high flexibility, low cost, low power consumption, and simple fabrication, this MOF-based MMM presents a promising platform for practical toxic gas monitoring in industrial applications.

Moreover, there was a study about a novel approach for fabricating flexible NO gas sensors by integrating UiO-66-NH_2_ MOFs into textiles using electrohydrodynamic jet (e-jet) printing. To enhance electrical conductivity and sensing performance, UiO-66-NH_2_ MOFs were combined with the IL 1-ethyl-3-methylimidazolium trifluoromethanesulfonate, forming a MOF-IL ink that was precisely printed onto polylactic acid (PLA) films [164]. The incorporation of IL significantly improved the MOF’s conductivity, achieving an approximately 14-fold enhancement, and resulted in an exceptionally high and reversible response to NO gas, with a conductance change of 1634.67%, far surpassing the negligible response of pristine MOF sensors. The sensing mechanism was influenced by moisture-assisted proton transport and thermally activated conduction, as confirmed by an activation energy of 114 meV. This work highlights the potential of e-jet-printed MOF-based textile sensors as a scalable and flexible platform for toxic gas detection, environmental monitoring, and safety applications.

Overall, polymer hybrids score high in scalability and adaptability, especially for wearable formats. The drawbacks are mostly around slower recovery times and possible polymer degradation in humid or harsh environments. ILs solve one problem, conductivity, while introducing another: stability over long cycles. Still, they remain an attractive direction for low-power, flexible sensing devices.

#### 4.3.3. Multi-Component and MOF-Derived Hybrids

Some researchers are not satisfied with just two components. By blending MOFs with conductive nanomaterials, polymers, and catalytic dopants all at once, they aim for “best of all worlds” systems.

Take MOF-derived tungsten ethoxide/polypyrrole-rGO composites, for example [165]. The sensor material was synthesized via a simple hydrothermal process, where polypyrrole–reduced graphene oxide (Ppy-rGO) was integrated with tungsten ethoxide as an organic linker, forming MOF-derived nanocrystals through hydrogen bonding. The synergistic combination of tungsten ethoxide and Ppy-rGO enhances the structural stability of the sensor, enables efficient detection of analytes at ambient temperature, and provides multiple electron and ion transport pathways for improved sensing performance. The detection mechanism is governed by the interaction between NH_4_^+^ ions and the MOF-derived framework, where increasing NH_4_^+^ concentration leads to higher proton (H^+^) generation, which strengthens bonding activity and improves electrical conductivity. Cyclic voltammetry measurements within the potential window of −1.5 to 1.5 V exhibit a quasi-rectangular profile, confirming stable and consistent electronic and ionic transport. The sensor demonstrates remarkable detection capabilities over a dynamic range of 0.85–3.35 µM, with a limit of detection of 0.278 µM (9.74 ppb) and a limit of quantification of 0.843 µM (29.54 ppb). These results highlight the potential of MOF-derived hybrid nanostructures for real-time toxic ion detection and their promising applications in environmental monitoring and agricultural safety.

More generally, semiconducting metal oxides (SMOx), carbon-based materials, and polymers each face significant limitations. SMOx-based sensors generally require high operating temperatures, carbon-based sensors often suffer from poor gas selectivity and slow response times, and polymer-based sensors exhibit limited stability and selectivity [166]. To overcome these challenges, MOFs have emerged as promising candidates due to their intrinsic high porosity, tunable structures, and large specific surface areas, which enable enhanced gas adsorption and sensitivity. Integrating MOFs with other sensing materials can create synergistic effects that improve detection performance, response speed, and selectivity. Numerous studies have explored MOF-based composite systems, often incorporating three or more components, to achieve optimized sensing characteristics. This review summarizes recent progress in the design, synthesis, and application of MOF-based composites for gas sensing, while also addressing current challenges and highlighting future directions for developing high-performance MOF-integrated gas sensors.

The obvious downside is synthesis complexity. Multi-component systems can be difficult to reproduce consistently, and costs rise quickly as more materials and processing steps are added. Scaling these hybrids to industrial levels may be unrealistic without simplification. But the promise is strong: they could unlock sensors that do not just work in the lab, but actually perform across diverse, real-world conditions. A concise overview of these comparative strengths and limitations, along with the advantages offered by MOFs, is provided in Table 3, which summarizes their potential for toxic gas detection.

### 4.4. Advantages of MOF-Based Sensors over Traditional Sensor Materials

To provide a clear comparison of MOF-based gas sensors with traditional sensor materials, Table 4 summarizes the key advantages, limitations, and performance characteristics of each class. While conventional materials such as metal oxide semiconductors (MOS), electrochemical sensors, and optical systems have long been employed for environmental monitoring, they often face trade-offs in sensitivity, selectivity, power consumption, and stability under real-world conditions [167,168,169,170]. In contrast, MOF-based sensors, particularly when integrated with nanostructures or hybrid composites, demonstrate enhanced room-temperature operation, tunable selectivity, and compatibility with low-power, miniaturized devices [24,80,171,172,173]. This table highlights how the structural and chemical versatility of MOFs positions them as a promising next-generation platform for efficient, reliable, and adaptable toxic gas detection.

## 5. Integration of MOF-Based Sensors with Embedded Systems and AI

The growing demand for real-time monitoring of toxic gases in industrial, urban, and confined settings has pushed MOF-based sensors toward integration with microelectronics, IoT networks, and AI-assisted analytics. These systems aim to combine high sensitivity and selectivity with portability and low-power operation, although deploying them under fluctuating or harsh conditions still presents practical challenges.

### 5.1. Plasmonic MOFs for Real-Time Sensing and AI-Assisted Analysis

Plasmonic MOFs have emerged as a highly promising class of materials for on-site, real-time detection, complementing traditional MOF-derived semiconductor and hybrid systems [174]. These composites integrate plasmonic metal nanoparticles, such as Au or Ag, within a MOF matrix, preserving the intrinsic porosity and chemical functionality of the MOF while introducing strong localized surface plasmon resonance (LSPR) effects [175]. The resulting synergistic combination enhances light matter interactions, electron transfer kinetics, and surface-enhanced Raman scattering (SERS) signals, enabling sensitive detection of chemical and biological analytes at trace concentrations [176].

Recent developments demonstrate the potential of flexible plasmonic MOF films, such as AgNWs@ZIF-8, which couple molecular capture capabilities with deep learning models to achieve classification accuracies exceeding 93% for gaseous aldehydes [177]. This highlights their applicability for portable environmental and medical sensing platforms. The integration of AI and machine learning further enables real-time spectral data processing, pattern recognition, and quantitative prediction, substantially improving sensor selectivity, sensitivity, and operational robustness under complex or fluctuating conditions. These advancements reflect the rapid growth of plasmonic MOFs and their significant potential for next-generation, intelligent, on-site MOF-based sensors.

Among these technologies, SERS-based plasmonic MOF platforms have shown particular promise for gas detection, even for molecules with low Raman cross sections. A notable example is a thin-film system composed of close-packed core–shell Au@Ag nanorods encapsulated within a ZIF-8 framework (Au@Ag@ZIF-8) [178]. In this platform, the plasmonic Au@Ag nanoparticles amplify Raman signals of adsorbed molecules, while the ZIF-8 MOF serves both as a selective adsorption medium and a Raman internal standard, facilitating preconcentration of target gases such as dimethyl methylphosphonate (DMMP) and 2-chloroethyl ethyl sulfide (CEES). Computational studies have elucidated the adsorption mechanisms within the ZIF-8 pores and the specific interactions between DMMP molecules and the Ag surface.

The Au@Ag@ZIF-8 thin films exhibit excellent SERS performance, including rapid response, low detection limits, high reproducibility, and recyclability. For example, a limit of detection (LOD) of 0.2 ppbV was achieved for DMMP. Portable Raman measurements demonstrated practical applicability, detecting 2.5 ppmV DMMP in ambient air and 76 ppbV CEES in N_2_, with response times of 21 s and 54 s, respectively. This platform establishes a proof-of-concept for handheld plasmonic MOF-based SERS gas sensors, enabling ultralow-level detection suitable for homeland security, chemical process monitoring, critical infrastructure protection, and personalized diagnostic applications [179].

### 5.2. Portable and Wearable MOF-Based Sensor Devices

Portable MOF-based platforms now pair sensor arrays with microcontrollers and wireless modules to enable continuous, on-site monitoring of multiple hazardous gases. For example, an Arduino UNO R3 system integrated with MOF-enhanced sensors AQ3, Minipid 2 HS PID, IR5500, MQ3, and DHT11 used an ESP8266 Wi-Fi module to transmit data to a cloud-based monitoring platform [180]. The system leveraged hybrid machine learning models combining Hidden Markov Models (HMM) with Artificial Neural Networks (ANN) to maintain fault-tolerant operation. Interestingly, this combination reduced false positives to as low as 0.01%, a substantial improvement over conventional monitoring methods.

Single-sensor systems tend to stumble in real-world environments, so some groups have gone for multimodal AI setups. In one case, researchers worked with 6400 gas samples and paired a semiconductor sensor array with a thermal imaging camera. Instead of treating each stream separately, they merged the two through an “early fusion” AI framework. The fused model clocked in at 96% accuracy, a clear jump over the LSTM-only (82%) and CNN-only (93%) approaches [181]. These results demonstrate that multimodal AI fusion significantly enhances gas detection performance compared to single-sensor approaches.

### 5.3. Flexible Substrates, Microelectronic Integration

**Figure 12:**
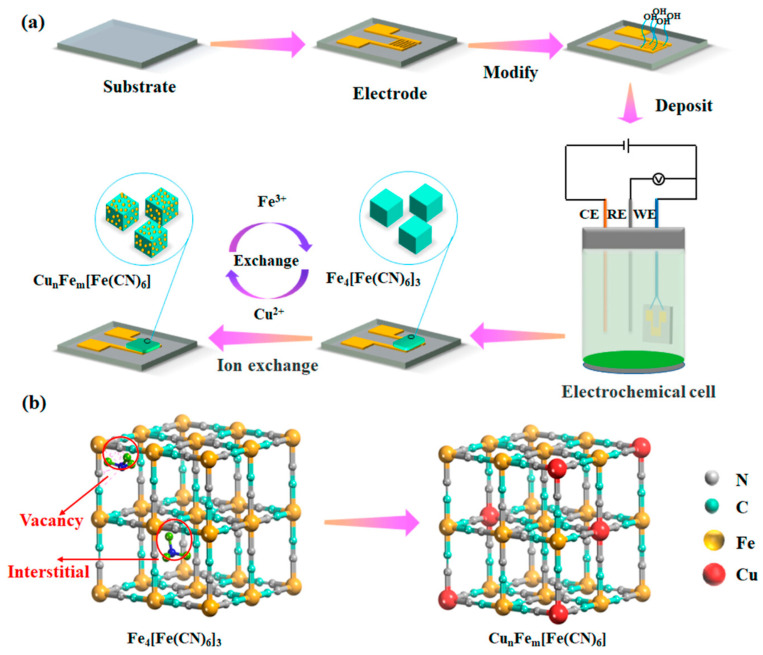
(**a**) Stepwise fabrication of the Cu–Fe PBA-based capacitive sensor on Au interdigitated electrodes (IDEs), including surface modification, PB film deposition, and ion-exchange to form Cu–Fe PBAs. (**b**) Crystal structures of PB and Cu–Fe PBAs, highlighting adsorption sites for ammonia gas. Reproduced with permission from ACS [182].

Another thread of research is heading in a slightly different direction: making the devices smaller, bendable, and easier to integrate into electronics we already use. Hou et al., for instance, built a capacitive ammonia sensor from a Cu–Fe Prussian blue analogue (PBA)-derived MOF [182]. The preparation steps and structural features of the Cu–Fe PBA-based capacitive sensor are shown in Figure 12a,b. This modification increased the specific surface area and adsorption sites while preserving the original PB framework. The resulting Cu–Fe PBA membrane was integrated with interdigitated electrodes (IDEs) to construct a capacitive sensor, where the sensitive film was optimized using hydrothermal synthesis and cyclic voltammetry deposition. Adsorption studies using QCM confirmed a high uptake capacity (~2.2 mmol g^−1^) and efficient ammonia trapping under ambient pressure. Structural and morphological analyses further validated the enhanced porosity and stability of the material. The final capacitive sensor demonstrated a linear response across 75–1000 ppb, with a detection limit as low as 3.8 ppb, highlighting the advantages of MOF–microelectrode integration for developing compact, ultrasensitive ammonia detection devices suitable for next-generation monitoring systems.

### 5.4. Advanced MOF-Based Materials for Trace Gas Detection

Beyond integration, material innovations have played a key role in achieving ultralow-level detection. One approach involves 0D–2D heterostructures, such as cobalt-based MOFs self-assembled on Ti_3_C_2_T_x_ MXene nanosheets [183]. The Co-MOF, with its high specific surface area, efficiently captures and concentrates target gas molecules, enhancing host–guest interactions and improving both selectivity and sensitivity. The highly conductive MXene nanosheets facilitate rapid electron transport across the heterointerface, accelerating reaction kinetics. Consequently, the resulting chemiresistive Co-MOF@MXene sensor exhibits outstanding sensitivity even in the presence of interfering gases, achieving a response value of 11.1 to 400 ppb H_2_S at RT. This rational design of MOF@MXene heterostructures paves the way for developing advanced hybrid materials for high-performance gas sensing applications.

A follow-up study confirmed that these Co-MOF@MXene heterostructures could reliably detect ppb-level H_2_S at room temperature even in the presence of interfering gases [184]. The work suggests that carefully designed MOF–MXene interfaces may be a promising route for portable, high-performance sensors, though one must remain mindful of potential reproducibility challenges when scaling up these heterostructures.

Other MOF-derived systems further illustrate the versatility of these materials. Fiber-optic sensors integrating Fabry–Perot interferometers with HKUST-1 MOFs detected ethanol and benzene with high sensitivity, and crystal polishing enhanced spectral stability and response times [185]. Testing with ethanol and benzene gases caused measurable shifts in the FPI interference signal. The sensor exhibited high sensitivity, detecting ethanol gas concentrations (EGCs) with 0.428 nm/ppm in the 24.9–40.11 ppm range and benzene gas concentrations (BGCs) with 0.15 nm/ppm in the 99–124 ppm range. Selectivity tests with ultralow concentrations of ethanol, benzene, and toluene revealed enhancement factors of 436% for benzene and 140% for toluene, attributed to improved miscibility of these conjugated-ring molecules with the ethanol-modified HKUST-1 framework.

In another study, a MEMS-based H_2_S sensor was developed using novel ZnCu-based oxide semiconductors derived from MOFs. To enhance gas sensing performance, two strategies were employed: heteroatom doping and heterogeneous interface engineering. The results revealed that sensors based on CuO/ZnO heterostructures exhibited unstable recovery behavior due to multiple interface formations after H_2_S adsorption and reaction [186]. In contrast, the ZnCu-MOS-3 sensor (Cu-doped ZnO) demonstrated superior performance, delivering a high response of 16.19 to 20 ppm H_2_S, a fast response time of 33 s, and autonomous recovery capability. Furthermore, a real-time H_2_S alarming platform was integrated with the sensor to prevent potential human exposure and poisoning. This work highlights a rational design strategy for developing efficient, MOF-derived semiconductor-based gas sensors, offering a promising approach for portable environmental monitoring and toxic gas safety applications.

MOF-derived semiconductor nanostructures, including In_2_O_3_/ZnO hollow nanocages templated from ZIF-8, exhibited markedly improved H_2_S detection at 200 °C, attributed to n-n heterojunction formation and porous morphology supporting gas diffusion [187]. In/Zn-ZIF-8 template material was synthesized by a simple one-step co-precipitation method followed by thermal annealing in air. The heat treatment resulted in In_2_O_3_/ZnO nanostructures with mixed heterostructures. The as-prepared In_2_O_3_/ZnO sensitive material had a microstructure of porous hollow nanocages with an average particle size of about 200 nm, which is beneficial to the diffusion and adsorption of gas molecules. The gas sensing performance test results of the In_2_O_3_/ZnO hollow nanocages show that their response to H_2_S gas is significantly improved, 67.5 @50 ppm H_2_S (about 11 times that of pure ZnO nanocages), at an optimal temperature of 200 °C, showing better selectivity, lower theoretical detection limit and good linearity between gas concentration and response values. The enhanced gas sensing feat to H_2_S gas is mainly attributed to the formation of n-n heterojunction and the wide surface area of the newly formed In_2_O_3_/ZnO porous hollow nanocages.

Flexible SERS-active MOF films, such as AgNWs@ZIF-8, combined molecular capture with deep learning, achieving 93.7% accuracy in classifying gaseous aldehydes, an encouraging result for portable environmental and medical sensing applications [188]. Under optimized structural parameters, the flexible film demonstrated excellent SERS performance for gaseous analytes, including 4-aminothiophenol, 4-mercaptophenol, and dithiohydroquinone, maintaining reproducible and stable Raman signals for up to 30 days. Furthermore, deep learning techniques were applied to classify SERS spectra obtained from mixed gaseous analytes within the MOF-based film. The integration of flexible MOF-based SERS sensors with AI-driven data analysis opens up exciting opportunities for noninvasive medical diagnostics, food safety monitoring, environmental analysis, and real-time portable sensing applications.

Low-dimensional MOFs, enhanced with stimuli like light or voltage modulation, further improve room-temperature sensitivity and selectivity [189]. Their unique structural, electronic, mechanical, and optical properties enable superior sensitivity and selectivity under RT conditions. This review highlights the latest developments in RT gas sensing technologies based on 0D, 1D, and 2D nanostructures, along with innovative external stimuli methods such as voltage modulation and light activation, which improve sensing performance without requiring high operating temperatures. Finally, the discussion extends to emerging device applications, including wearable gas sensors, AI-driven data analytics, machine learning-assisted detection, and neuromorphic olfactory systems, while outlining current challenges and future opportunities in developing portable, intelligent gas sensing platforms based on low-dimensional nanomaterials and MOFs.

Each material class presents inherent trade-offs. Conventional sensors offer durability and low cost but often fall short in sensitivity and selectivity. Carbon nanomaterials and polymers provide tunability and flexibility, yet their performance can degrade under harsh conditions. MOF-based hybrids demonstrate superior selectivity and effective room-temperature operation, though challenges remain in device integration and long-term stability.

## 6. Challenges and Future Directions

### 6.1. Technical Challenges

#### 6.1.1. Reproducibility and Long-Term Stability

MOS have historically dominated toxic gas sensing because they are inexpensive and relatively durable. Yet their high operating temperatures, limited selectivity, and vulnerability to cross-interference leave them ill-suited for the increasingly complex requirements of environmental monitoring [190]. MOFs, by contrast, offer tunable porosity, diverse chemistry, and room-temperature operation, all of which directly address the shortcomings of MOS platforms. Still, practical deployment of MOF-based sensors is hindered by issues of reproducibility and stability [191]. Small variations in synthesis often alter particle morphology or defect density, leading to inconsistent performance. Progress in water-stable MOF design and systematic aging studies will be critical to move MOFs from the laboratory into reliable field applications.

#### 6.1.2. Standardization of Fabrication and Device Interfaces

While MOS sensors benefit from decades of industrial refinement, MOF-based devices remain at an early stage of integration. Current fabrication strategies, such as drop-casting, layer-by-layer assembly, or MOF chemical vapor deposition, frequently yield variations in film quality, adhesion, and conductivity [192]. Similarly, the lack of standardized testing protocols complicates direct comparison across studies. For MOFs to transition from proof-of-concept to scalable technologies, reproducible fabrication methods and well-defined benchmarking standards must be established [193]. Drawing from semiconductor industry practices, greater attention to process standardization will strengthen the reliability of MOF–device interfaces and accelerate their translation to commercial sensors.

### 6.2. Data Management and Cybersecurity

Unlike MOS devices, which generate relatively simple datasets, MOF-based arrays often produce complex multidimensional signals due to their high sensitivity and selectivity. This creates opportunities for advanced analytics but also raises challenges in secure storage, transmission, and interpretation of data [194]. The integration of machine learning provides a powerful tool for extracting meaningful insights from MOF sensor outputs; however, reliance on data-driven models introduces risks of bias, lack of transparency, and vulnerability to cyberattacks [191,195]. Ensuring secure communication protocols and incorporating explainable AI frameworks will be essential for building trust in MOF-based sensing networks, particularly in industrial and healthcare settings.

### 6.3. Opportunities for Next-Generation Environmental Monitoring

#### 6.3.1. IoT-Enabled MOF Sensor Networks

The inherent room-temperature operation of many MOFs positions them as ideal candidates for low-power Internet-of-Things (IoT) applications, in contrast to MOS sensors that typically require heating [196]. Wireless MOF-based sensor networks could enable real-time, high-resolution mapping of pollutants in industrial complexes, transportation corridors, and urban centers [197]. Coupling MOFs with low-power communication technologies such as LoRa or NB-IoT opens pathways for scalable and energy-efficient deployment [198].

#### 6.3.2. Multi-Analyte Detection and Selectivity Engineering

Cross-sensitivity remains one of the most persistent limitations of MOS sensors, often leading to false positives in mixed-gas environments. MOFs offer a structural toolkit for overcoming this challenge. Through precise control of pore size, functional groups, and metal centers, MOFs can be engineered to discriminate between chemically similar gases. Hybrid designs that integrate MOFs with conductive nanocarbons or polymers extend this advantage further, enabling simultaneous detection of multiple analytes, a capability critical for environmental monitoring and health diagnostics.

#### 6.3.3. AI-Assisted Adaptive Monitoring Systems

The complexity of MOF sensing signals provides fertile ground for artificial intelligence-driven interpretation. Unlike traditional MOS sensors, which are typically limited to threshold-based responses, MOF–AI systems can adapt to environmental fluctuations, correct for humidity interference, and even predict sensor degradation [199,200]. Early demonstrations indicate that MOF–AI platforms outperform conventional systems in accuracy and resilience, pointing toward the development of adaptive monitoring tools that go beyond detection to deliver real-time environmental insights.

### 6.4. Balancing Challenges and Opportunities

In summary, while MOS materials remain practical and established, their fundamental limitations, selectivity, power demand, and adaptability make them less suited for next-generation sensing demands. MOFs, with their tunable chemistry, structural diversity, and compatibility with room-temperature operation, present a compelling alternative. The key challenges of reproducibility, stability, and standardization should not be understated, but they are not insurmountable. With continued advances in water-stable MOFs, reproducible fabrication, and AI-enabled analytics, MOF-based sensors are positioned to evolve from promising research tools into transformative platforms for toxic gas detection and environmental monitoring.

## 7. Conclusions and Outlooks

The MOFs are a paradigm set of gas sensor materials with uniquely high porosity, chemically adjustable surface area, and modularity, which are difficult to achieve with traditional sensor materials. The inherent structural attributes of tunable pore size, open-metal sites, and functionalization permit host–guest interaction to be controlled to an unprecedented extent to allow selective trapping of desired gases at trace levels. The drawings referred to throughout this review show how functional group substitutions, incorporation of noble metals, and hybridization with conjugated polymers or conductive dopants can greatly improve sensitivity, selectivity, and stability. The modularity and post-synthetic functionalization of MOFs are especially desirable properties for incorporation of catalytic, luminescent, and redox-active functionality optimized for a desired sensing mission. MOF nanostructures and composites based thereon further enhance this versatility by offering added stability, conductivity, and dynamic response functionality. These advances link theoretical MOF properties and practical sensor performance by connecting materials design and system application.

Also critical are the transduction processes facilitated by MOFs. Resistive/electrical sensing is by far the most explored, whereby gas adsorption leads to measurable conductivity or resistance variations. Though inherent MOFs are frequently constrained by low conductivity, MOF–carbon composite materials, oxide materials derived by MOFs, and defect engineering have overcome this issue and yielded extremely responsive and selective chemiresistive materials. In resistive modes aside from those, QCM- and photoluminescence-based MOF sensors show outstanding VOC-, toxic gas- (e.g., SO_2_, HF, H_2_S), and CO_2_-detecting capacities, and further enhance the range of application for detection technology empowered by MOFs.

In spite of these brighter prospects, some challenges still lie ahead for commercialization of the MOF-based sensors:

- Scale and reproducibility of MOF synthesis and incorporation into device structures continue to be non-trivial.
- Long-term stability under real-world conditions (humidity, temperature fluctuations, chemical interferences) must be ensured.
- Signal transduction has to be enhanced significantly, especially for room-temperature resistive sensors, to challenge already established semiconducting oxides.
- Selectivity in complex matrices is an enduring challenge to be addressed by using increasingly sophisticated methods such as MOF electronic noses or multi-sensor arrays.

Looking forward, several avenues are poised to advance the field:

- Rational design of multifunctional MOFs through defect engineering, heteroatom doping, and modular linker strategies to optimize porosity, binding affinity, and charge transport simultaneously.
- The incorporation of high-end transduction platforms such as photonic devices, plasmonic sensors, and soft electronics is aimed at enhancing detection modes and portability.
- Artificial Intelligence (AI) and Machine Learning (ML) integration, where AI/ML algorithms can decode complex sensor array outputs (e.g., MOF electronic noses) for highly accurate discrimination of VOC isomers, pollutant blends, and disease biomarkers. Supervised learning, neural networks, and hybrid chemometric approaches allow the extraction of subtle patterns in multidimensional MOF sensor datasets, improving sensitivity, selectivity, and real-time decision-making capabilities.
- Ecological and green fabrication methods such as inkjet printing, electrospinning, or MOF-CVD to develop wearable, flexible, and low-power sensor devices.

Application-driven engineering is all about application-specific tuning of MOF sensors to desired applications like environmental safety, industrial monitoring, healthcare diagnostics, or high-end automotive safety applications. Quite simply, key synergy between intrinsic MOF properties, functional modularity by design, and multi-dimensional transduction schemes has made MOFs a foundation upon which next-generation gas detection technology will improve. With continued innovation in functionalization schemes, device incorporation, and data-driven modeling, MOF-based sensors have tremendous potential to address a host of challenges plaguing environmental monitoring, public health, and safety-critical communities.

## Figures and Tables

**Table 1:** Maximum allowable indoor air concentrations and health relevance of major toxic gases and VOCs relevant to environmental and gas-sensing applications.

Pollutant/VOC	Common Source	Health Impact	Maximum Indoor Air Concentration	Guideline
Formaldehyde	Furniture, building materials	Irritation, carcinogenic	0.1 mg/m^3^ (WHO, 30 min avg)	WHO (2010)
Benzene	Paints, tobacco smoke	Carcinogenic	5 µg/m^3^ (EU Directive 2000/69/EC)	EU
Toluene	Adhesives, solvents	Nervous system effects	260 µg/m^3^ (WHO, 1-week avg)	WHO
CO	Incomplete combustion	Headache, dizziness	10 mg/m^3^ (8 h avg, WHO)	WHO
NO_2_	Gas stoves, heaters	Respiratory irritation	200 µg/m^3^ (1 h avg, WHO)	WHO
O_3_	Photochemical reactions	Lung irritation	100 µg/m^3^ (8 h avg, WHO)	WHO
Total VOCs	Various indoor sources	Eye/nose irritation	<300 µg/m^3^ (recommended)	ISO 16000-6

**Table 2:** Summary table on source and environmental impact of toxic gas.

Gas	Primary Sources	Health Impacts	Environmental Impacts	MOF/Sensor	Notes	Ref.
NO_x_	Vehicles, industrial facilities, power plants	Respiratory issues, worsened asthma, cardiovascular problems	Acid rain, smog, soil and water acidification, vegetation stress, ecosystem imbalance	La-doped ZnO porous nanocages encapsulated in MOF	Fast response 28 s; detection limit 5.68 ppb NO_2_; stable under humidity; high selectivity	[46]
SO_2_	Fossil fuel combustion, industrial operations, power generation	Respiratory irritation	Forms acid rain; damages soil, water, forests, crops; alters aquatic ecosystems	MOFs for selective SO_2_ adsorption	Potential for trapping industrial SO_2_; improves environmental mitigation	[47]
CO	Industrial emissions, vehicles, biomass burning	Interferences with oxygen transport; acute poisoning risk	Affects ecosystems; partially mitigated by microbial CO oxidation	MOFs for CO adsorption	Enhances detection sensitivity and selectivity; can operate in varying humidity	[48]
O_3_	Photochemical reactions between NO_x_ and VOCs	Lung and eye irritation	Damages vegetation; contributes to smog and acid rain	–	Sensor platforms required for fast detection in outdoor air	[49]
VOCs	Industrial activities, chemical processing, stationary sources	Respiratory issues, neurological effects, cancer risk	Air pollution, smog, ecosystem imbalance	MOF-based fluorescent/ratiometric sensors	Can detect benzene, formaldehyde, vinyl chloride, 1,3-butadiene; portable and selective	[50]
NH_3_	Fertilizer application, livestock management	–	Contributes to ecosystem imbalance	MOFs for NH_3_ adsorption	Enhances gas capture and monitoring in agricultural settings	[57]
N_2_H_4_	Industrial chemical processes	Highly toxic, environmental hazard	Pollutants in water, soil, and air	DIPOT ratiometric fluorescent sensor	Detection limit 4.5 nM; visible fluorescence shift; portable smartphone-read test strips; validated in 20 samples	[68]

**Table 3:** Representative MOF-based sensing materials and their gas detection performance.

Materials	Target Analyte Gas	Method	LOD (ppb)	Linear Range (ppb)	Ref.
UiO-66–NH_2_/P3HT hybrid film	NO_2_	Resistive	1 × 10^−6^	–	[94]
NH_2_–MIL-101(Cr)/QCM	HF	QCM	500	–	[97]
ZnFe_2_O_4_/rGO (MOF-derived)	NO_2_	Resistive	0.149	50–4000	[107]
Co_3_O_4_ nanosheets (MOF-derived)	H_2_S	Resistive	500	500–100,000	[109]
Co-doped ZnO/ZIF hybrid	H_2_S	Resistive	70	–	[110]
(Hf)PCN-224(Co)	SO_2_	Photoluminescence	175,500	–	[120]
UiO-66-NH_2_ nanofibers/CNT	SO_2_	Capacitive	1000	1000–125,000	[123]
MOF-e-nose (array of MOFs on QCM)	VOC (xylene)	QCM	1000	100,000	[126]
MUF-16	SO_2_	Fluorescence quenching	80,720	-	[135]
SIFSIX-1-Cu	SO_2_	Microwave dielectric sensing	8.9	10–1,000,000	[141]
Rh6G@UiO-66-NH_2_	NO_2_^−^	Ratiometric fluorescence	0.966	46–4600	[142]
Ni–Mg MOF-74 films	NO_2_	Impedance	1000	-	[144]
PABA@MOF-808	NO	Fluorescence quenching	21	-	[146]
CoNiHHTP MOF/PHI heterojunction	NO_2_	Optoelectronic	1	-	[147]
Ru@MOF-NH_2_	NO_2_^−^	Ratiometric fluorescence + smartphone readout	27.6	-	[148]

**Table 4:** Comparative overview of traditional sensor materials and MOF-based systems for toxic gas detection.

Sensor Material	Strengths	Limitations	MOF-Based Advantage	Notes
Metal Oxide Semiconductors (MOS)	Robust, low-cost, sensitive	Robust, low-cost, sensitive	Robust, low-cost, sensitive	Robust, low-cost, sensitive
Electrochemical Sensors	Compact, low-cost, portable	Compact, low-cost, portable	Compact, low-cost, portable	Compact, low-cost, portable
Optical Sensors	High precision, stable baseline	High precision, stable baseline	High precision, stable baseline	High precision, stable baseline
Carbon Nanomaterials (Graphene, CNTs)	High surface area, conductivity, functionalization	High surface area, conductivity, functionalization	High surface area, conductivity, functionalization	High surface area, conductivity, functionalization
Conductive Polymers	Low-cost, flexible, tunable doping	Low-cost, flexible, tunable doping	Low-cost, flexible, tunable doping	Low-cost, flexible, tunable doping
MOF-Based Hybrids	Ambient operation, tunable, multi-gas selectivity	Ambient operation, tunable, multi-gas selectivity	Ambient operation, tunable, multi-gas selectivity	Ambient operation, tunable, multi-gas selectivity

## Data Availability

No new data were created or analyzed in this study.

## Abbreviations

The following abbreviations are used in this manuscript:

NO_x_	nitrogen oxides
SO_2_	sulfur dioxide
CO	carbon monoxide
O_3_	ozone
VOCs	volatile organic compounds
IR	infrared
UV	ultraviolet
QCM	quartz crystal microbalance
MOS	metal oxide semiconductor
MOFs	metal–organic frameworks
AI	artificial intelligence
CO_2_	carbon dioxide
H_2_S	hydrogen sulfide
La	lanthanum
ZnO	zinc oxide
SnO_2_	tin dioxide
WO_3_	tungsten trioxide
NH_3_	ammonia
N_2_H_4_	hydrazine
FET	field-effect transistor
MoS_2_	molybdenum disulfide
CH_4_	methane
H_2_O	water
CuNCs	copper nanoclusters
CNQDs	nitrogen-doped carbon quantum dots
g-C_3_N_4_	graphitic carbon nitride
NaOH	sodium hydroxide
h-MoO_3_	hexagonal molybdenum trioxide
TiO_2_	titanium dioxide
R_a_/R_g_	resistance ratio
TMDCs	transition metal dichalcogenides
ppb	parts per billion
μM	micromolar
ppt	parts per trillion
MMM	mixed-matrix membrane
IL	ionic liquid
CS	chitosan
PVA	polyvinyl alcohol
NO	nitric oxide
UiO-66-NH_2_	amino-zirconium-based MOF
e-jet	electrohydrodynamic jet
FTIR	attenuated total reflectance–Fourier transform infrared
Ppy-rGO	polypyrrole–reduced graphene oxide
NH_4_^+^	ammonium ion
H^+^	proton
SMOx	semiconducting metal oxides
P3HT	poly(3-hexylthiophene)
CNT	carbon nanotubes
NO_2_	nitrogen dioxide
Ru	ruthenium
PB	Prussian blue
IDE	interdigitated electrode
HMM	hidden Markov model
ANN	artificial neural network
LSTM	long short-term memory
CNN	convolutional neural network
PBA	Prussian blue analogue
RT	room temperature
Ti_3_C_2_T_x_	MXene nanosheet
Co-MOF	cobalt-based MOF
FPI	Fabry–Perot interferometer
EGC	ethanol gas concentration
BGC	benzene gas concentration
ZnCu-MOS Zn/Cu	metal oxide semiconductor
In_2_O_3_	indium oxide
ZIF-8	zeolitic imidazolate framework-8
AgNWs	silver nanowires
SERS	surface-enhanced Raman spectroscopy

## References

[1] Cruz-martínez H., Rojas-chávez H., Montejo-alvaro F., Peña-castañeda Y.A., Matadamas-ortiz P.T., Medina D.I. (2021). Recent developments in graphene-based toxic gas sensors: A theoretical overview. Sensors.

[2] Benedetto G., Mirica K.A. (2024). Conductive Framework Materials for Chemiresistive Detection and Differentiation of Toxic Gases. Accounts Chem. Res..

[3] Rezaei I., Haghverdi A.B., Soldoozy A., Aghaee T., Biabanifard S. (2024). Wearable Kapton graphene biosensor for detection of toxic gases. J. Hazard. Mater. Adv..

[4] David E., Niculescu V.C. (2021). Volatile organic compounds (Vocs) as environmental pollutants: Occurrence and mitigation using nanomaterials. Int. J. Environ. Res. Public Health.

[5] Aldalbahi A., El-Naggar M.E., El-Newehy M.H., Rahaman M., Hatshan M.R., Khattab T.A. (2021). Effects of technical textiles and synthetic nanofibers on environmental pollution. Polymers.

[6] Lasek J.A., Lajnert R. (2022). On the Issues of NOx as Greenhouse Gases: An Ongoing Discussion. Appl. Sci..

[7] Dhall S., Mehta B.R., Tyagi A.K., Sood K. (2021). A review on environmental gas sensors: Materials and technologies. Sens. Int..

[8] Hannun R.M., Razzaq A.H.A. (2022). Air Pollution Resulted from Coal, Oil and Gas Firing in Thermal Power Plants and Treatment: A Review. IOP Conf. Ser. Earth Environ. Sci..

[9] Ettlinger R., Lächelt U., Gref R., Horcajada P., Lammers T., Serre C., Couvreur P., Morris R.E., Wuttke S. (2022). Toxicity of metal-organic framework nanoparticles: From essential analyses to potential applications. Chem. Soc. Rev..

[10] Menon D., Chakraborty S. (2023). How safe are nanoscale metal-organic frameworks?. Front. Toxicol..

[11] Awewomom J., Dzeble F., Takyi Y.D., Ashie W.B., Ettey E.N.Y.O., Afua P.E., Sackey L.N.A., Opoku F., Akoto O. (2024). Addressing global environmental pollution using environmental control techniques: A focus on environmental policy and preventive environmental management. Discov. Environ..

[12] Zhao D., Yu S., Jiang W.J., Cai Z.H., Li D.L., Liu Y.L., Chen Z.Z. (2022). Recent Progress in Metal-Organic Framework Based Fluorescent Sensors for Hazardous Materials Detection. Molecules.

[13] Cho S.H., Suh J.M., Eom T.H., Kim T., Jang H.W. (2020). Colorimetric Sensors for Toxic and Hazardous Gas Detection: A Review. Electron. Mater. Lett..

[14] Allsop T., Neal R. (2021). A review: Application and implementation of optic fibre sensors for gas detection. Sensors.

[15] Alanazi N., Almutairi M., Alodhayb A.N. (2023). A Review of Quartz Crystal Microbalance for Chemical and Biological Sensing Applications.

[16] Sun L., Rotaru A., Robeyns K., Garcia Y. (2021). A Colorimetric Sensor for the Highly Selective, Ultra-sensitive, and Rapid Detection of Volatile Organic Compounds and Hazardous Gases. Ind. Eng. Chem. Res..

[17] Khoshnobish S.R., Ahmed T., Arefin T., Piya A.A., Shamim S.U.D. (2024). Assessing the sensing performance of Janus transition metal dichalcogenides (ScSSe, TiSSe and ZrSSe) for oxygencontaining toxic gas molecules such as CO, NO, NO_2_ and SO_2_. Appl. Surf. Sci..

[18] Ou W., Shen J., Zhong J., He J., Lei D., Li H., Chen Y., Wang C., Wu H., Zhou B. (2022). Rapid on-site detection of zinc pyrithione in real-life samples with unprecedented selectivity and sensitivity. Sens. Actuators B Chem..

[19] Bulowski W., Knura R., Socha R.P., Basiura M., Skibińska K., Wojnicki M. (2024). Thin Film Semiconductor Metal Oxide Oxygen Sensors: Limitations, Challenges, and Future Progress. Electronics.

[20] Goel N., Kunal K., Kushwaha A., Kumar M. (2022). Metal oxide semiconductors for gas sensing. Eng. Rep..

[21] Gorbova E., Tzorbatzoglou F., Molochas C., Chloros D., Demin A., Tsiakaras P. (2021). Fundamentals and principles of solid-state electrochemical sensors for high temperature gas detection. Catalysts.

[22] Fazio E., Spadaro S., Corsaro C., Neri G., Leonardi S.G., Neri F., Lavanya N., Sekar C., Donato N., Neri G. (2021). Metal-Oxide Based Nanomaterials: Synthesis, Characterization and Their Applications in Electrical and Electrochemical Sensors. Sensors.

[23] Sohrabi H., Maleki F., Khaaki P., Kadhom M., Kudaibergenov N., Khataee A. (2023). Electrochemical-Based Sensing Platforms for Detection of Glucose and H_2_O_2_ by Porous Metal–Organic Frameworks: A Review of Status and Prospects. Biosensors.

[24] Yuan H., Li N., Fan W., Cai H., Zhao D. (2021). Metal-Organic Framework Based Gas Sensors. Adv. Sci..

[25] Mehmandoust M., Çakar S., Özacar M., Salmanpour S., Erk N. (2021). Electrochemical Sensor for Facile and Highly Selective Determination of Antineoplastic Agent in Real Samples Using Glassy Carbon Electrode Modified by 2D-MoS_2_ NFs/TiO_2_ NPs. Top. Catal..

[26] Mphuthi N., Sikhwivhilu L., Ray S.S. (2022). Functionalization of 2D MoS_2_ Nanosheets with Various Metal and Metal Oxide Nanostructures: Their Properties and Application in Electrochemical Sensors. Biosensors.

[27] Nashruddin S.N.A.B.M., Salleh F.H.M., Yunus R.M., Zaman H.B. (2024). Artificial intelligence−powered electrochemical sensor: Recent advances; challenges; prospects. Heliyon.

[28] Kidanemariam A., Cho S. (2024). Recent Advances in the Application of Metal–Organic Frameworks and Coordination Polymers in Electrochemical Biosensors. Chemosensors.

[29] Chattopadhyay P.K., Singha N.R. (2021). MOF and derived materials as aerogels: Structure, property, and performance relations. Co-ord. Chem. Rev..

[30] Kidanemariam A., Pham D.T.T., Muhammad A., Min G., Cho S., Park J. (2025). Zn-H4dbp/GO for adsorptive removal and Zn-H4dbp/Au for electrochemical reduction of toxic Cr(VI) in aqueous solutions. J. Ind. Eng. Chem..

[31] Kidanemariam A., Cho S. (2025). Recent Advancements in Metal–Organic Framework-Based Microfluidic Chips for Biomedical Applications. Micromachines.

[32] Zuluaga S., Fuentes-Fernandez E.M.A., Tan K., Xu F., Li J., Chabal Y.J., Thonhauser T. (2016). Understanding and controlling water stability of MOF-74. J. Mater. Chem. A.

[33] Tang H., Fan D., Chen Y., Han S. (2025). Exploring enzyme-MOF (metal-organic framework) catalytic systems: Trade-offs between enzyme activity and MOF stability. Green Chem..

[34] Ahmadi M., Ayyoubzadeh S.M., Ghorbani-Bidkorbeh F., Shahhosseini S., Dadashzadeh S., Asadian E., Mosayebnia M., Siavashy S. (2021). An investigation of affecting factors on MOF characteristics for biomedical applications: A systematic review. Heliyon.

[35] Sun Q.J., Guo W.T., Liu S.Z., Tang X.G., Roy V.A.L., Zhao X.H. (2024). Rise of Metal-Organic Frameworks: From Synthesis to E-Skin and Artificial Intelligence. ACS Appl. Mater. Interfaces.

[36] Zhu J., Wen H., Fan Y., Yang X., Zhang H., Wu W., Zhou Y., Hu H. (2022). Recent advances in gas and environmental sensing: From micro/nano to the era of self-powered and artificial intelligent (AI)-enabled device. Microchem. J..

[37] Zhang Y., Liu H., Xu F. Research on the Application of Artificial Intelligence Technology in Microelectronics Testing and Fault Diagnosis. Proceedings of the 2024 3rd International Conference on Data Analytics, Computing and Artificial Intelligence (ICDACAI).

[38] Dastageer F., Areeckal A.S. (2025). On-Chip Integration of Micro-supercapacitor in VLSI Design for Power Management in Artificial Intelligence Processors and Memory Chips: A Review of Methods and Materials. Arab. J. Sci. Eng..

[39] Yang W., Qin Y., Wang Z., Yu T., Ge Z. (2022). Recent Advances in the Development of Flexible Sensors: Mechanisms, Materials, Performance Optimization, and Applications.

[40] Almassad H.A., Abaza R.I., Siwwan L., Al-Maythalony B., Cordova K.E. (2022). Environmentally adaptive MOF-based device enables continuous self-optimizing atmospheric water harvesting. Nat. Commun..

[41] Nieder R., Benbi D.K. (2023). Potentially toxic elements in the environment—A review of sources, sinks, pathways and mitigation measures. Rev. Environ. Health.

[42] Lebel E.D., Michanowicz D.R., Bilsback K.R., Hill L.A.L., Goldman J.S.W., Domen J.K., Jaeger J.M., Ruiz A., Shonkoff S.B.C. (2022). Composition; Emissions, and Air Quality Impacts of Hazardous Air Pollutants in Unburned Natural Gas from Residential Stoves in California. Environ. Sci. Technol..

[43] Siddiqua A., Hahladakis J.N., Al-Attiya W.A.K.A. (2022). An overview of the environmental pollution and health effects associated with waste landfilling and open dumping. Environ. Sci. Pollut. Res..

[44] Alamgholiloo H., Asgari E., Sheikhmohammadi A., Ghasemian N., Hashemzadeh B., Nourmoradi H. (2024). Enhancement of the catalytic performance of Co-ZIF/WO_3_ heterostructures for selective catalytic reduction of NOx. Sci. Rep..

[45] Deng J., Wang X., Wei Z., Wang L., Wang C., Chen Z. (2021). A review of NOx and SOx emission reduction technologies for marine diesel engines and the potential evaluation of liquefied natural gas fuelled vessels. Sci. Total. Environ..

[46] Panigrahi T.H., Sahoo S.R., Murmu G., Maity D., Saha S. (2022). Current challenges and developments of inorganic/organic materials for the abatement of toxic nitrogen oxides (NOx)—A critical review. Prog. Solid State Chem..

[47] Abdalla T.M., Adam M.A., Onaizi S.A. (2024). SO_2_ capture using nanostructured materials: Recent developments, challenges, and future outlooks. Surf. Interfaces.

[48] Rawat S., Bamola P., Rani C., Kaushik V., Kumar U., Dwivedi C., Rattan R., Sharma M., Kumar R., Sharma H. (2023). Interdigitated electrodes-based Au-MoS_2_ hybrid gas sensor for sensing toxic CO and NH_3_ gases at room temperature. Nanotechnology.

[49] Khatun R., Rocky M.H., Roy D., Al Roman A., Ahmed M.T. (2024). Ab initio study of Ti-doped C_3_N nanosheet as COCl_2_, O_3_, and HCN gas sensor. Comput. Theor. Chem..

[50] Tsai W.T. (2023). A Survey on Toxic Volatile Organic Compounds (VOCs): Toxicological Profiles, Health Exposure Risks, and Regulatory Strategies for Mitigating Emissions from Stationary Sources in Taiwan. Atmosphere.

[51] Pistollato F., Madia F., Corvi R., Munn S., Grignard E., Paini A., Worth A., Bal-Price A., Prieto P., Casati S. (2021). Current EU Regulatory Requirements for the Assessment of Chemicals and Cosmetic Products: Challenges and Opportunities for Introducing New Approach Methodologies.

[52] Brown S.K., Sim M.R., Abramson M.J., Gray C.N. (1994). Concentrations of Volatile Organic Compounds in Indoor Air—A Review. Indoor Air.

[53] Alberts W.M. (1994). Indoor air pollution: No, No_2_, CO, and CO_2_. J. Allergy Clin. Immunol..

[54] Chaloulakou A., Mavroidis I. (2002). Comparison of indoor and outdoor concentrations of CO at a public school. Evaluation of an indoor air quality model. Atmos. Environ..

[55] Schibuola L., Tambani C. (2020). Indoor environmental quality classification of school environments by monitoring PM and CO_2_ concentration levels. Atmos. Pollut. Res..

[56] Ozbay G., Jones M., Gadde M., Isah S., Attarwala T. (2021). Design and Operation of Effective Landfills with Minimal Effects on the Environment and Human Health. J. Environ. Public Health.

[57] Agache I., Sampath V., Aguilera J., Akdis C.A., Akdis M., Barry M., Bouagnon A., Chinthrajah S., Collins W., Dulitzki C. (2022). Climate change and global health: A call to more research and more action. Allergy.

[58] Zhang Y., Cui X., Xing H. (2021). Recent advances in the capture and abatement of toxic gases and vapors by metal-organic frameworks. Mater. Chem. Front..

[59] Popescu S.M., Mansoor S., Wani O.A., Kumar S.S., Sharma V., Sharma A., Arya V.M., Kirkham M.B., Hou D., Bolan N. (2024). Artificial intelligence and IoT driven technologies for environmental pollution monitoring and management. Front. Environ. Sci..

[60] Siddiqui J.A., Fan R., Naz H., Bamisile B.S., Hafeez M., Ghani M.I., Wei Y., Xu Y., Chen X. (2023). Insights into insecticide-resistance mechanisms in invasive species: Challenges and control strategies. Front. Physiol..

[61] Kibria M.G., Masuk N.I., Safayet R., Nguyen H.Q., Mourshed M. (2023). Plastic Waste: Challenges and Opportunities to Mitigate Pollution and Effective Management.

[62] Acharyya S., Nag S., Kimbahune S., Ghose A., Pal A., Guha P.K. (2021). Selective Discrimination of VOCs Applying Gas Sensing Kinetic Analysis over a Metal Oxide-Based Chemiresistive Gas Sensor. ACS Sens..

[63] Ji Y., Zhang N., Xu J., Jin Q., San X., Wang X. (2023). Co_3_O_4_/In_2_O_3_ p-n heterostructures based gas sensor for efficient structure-driven trimethylamine detection. Ceram. Int..

[64] Barik P., Pradhan M. (2022). Selectivity in trace gas sensing: Recent developments, challenges, and future perspectives. Analyst.

[65] Wawrzyniak J. (2023). Advancements in Improving Selectivity of Metal Oxide Semiconductor Gas Sensors Opening New Perspectives for Their Application in Food Industry. Sensors.

[66] Hussain S., Wang S., Amu-Darko J.N.O., Begi A.N., Yusuf K., Ibrahim T.K., Iqbal A., Manavalan R.K., Zhang X., Qiao G. (2024). MOF-derived La-doped ZnO dodecahedron nanostructures for efficient detection of NO_2_ gas. Sens. Actuators B Chem..

[67] Dennler N., Rastogi S., Fonollosa J., van Schaik A., Schmuker M. (2022). Drift in a popular metal oxide sensor dataset reveals limitations for gas classification benchmarks. Sens. Actuators B Chem..

[68] Li X.H., Li M.Z., Yang X.Y., Wang T.Y., Luo Y.H., Kandegama W.M.W.W., Li J.Y., Hao G.F., Liu C.R. (2025). Ultra-sensitive, versatile and portable detection of hydrazine in eco-environmental systems using a smartphone-integrated ratiometric fluorescent sensor. J. Hazard. Mater..

[69] Bhushan P., Kamat V., Abrol I., Kaushik A., Bhansali S. (2022). Bio-acceptability of wearable sensors: A mechanistic study towards evaluating ionic leaching induced cellular inflammation. Sci. Rep..

[70] Yun J., Cho M., Lee K., Kang M., Park I. (2022). A review of nanostructure-based gas sensors in a power consumption perspective. Sens. Actuators B Chem..

[71] Song P., Wang T. (2022). Application of Polyoxometalates in Chemiresistive Gas Sensors: A Review. ACS Sens..

[72] Ou L.X., Liu M.Y., Zhu L.Y., Zhang D.W., Lu H.L. (2022). Recent Progress on Flexible Room-Temperature Gas Sensors Based on Metal Oxide Semiconductor. Nano-Micro Lett..

[73] Yang B., Myung N.V., Tran T.T. (2021). 1D Metal Oxide Semiconductor Materials for Chemiresistive Gas Sensors: A Review. Adv. Electron. Mater..

[74] Sharma A., Eadi S.B., Noothalapati H., Otyepka M., Lee H.D., Jayaramulu K. (2024). Porous materials as effective chemiresistive gas sensors. Chem. Soc. Rev..

[75] Liu H., Zhang S., Cheng Q., Wang L., Wang S. (2023). A Mini Review on the Recent Progress of MoS_2_-Based Gas Sensors. Catal. Lett..

[76] Jiang T., He Q., Bi M., Chen X., Sun H., Tao L. (2021). First-principles calculations of adsorption sensitivity of Au-doped MoS_2_ gas sensor to main characteristic gases in oil. J. Mater. Sci..

[77] Borse R.G., Ingole S.M., Gaikwad S.S., Shinde V.S., Jadhav G.R., Gurule A.C., Dabhade G.B., Ghotekar S. (2025). Insights into a low-temperature gas sensing performance of hydrothermally fabricated novel Sn-modified LaCoO_3_ nanostructures. J. Mater. Sci. Mater. Electron..

[78] Wang G., Wang Y., Guo L., Chen T., Zhao W., Liu X., Wang J., Wang X., Yang Y. (2024). Chemiresistive n-butanol gas sensors based on Au@In_2_O_3_ hollow-sphere-array thin films. Sens. Actuators B Chem..

[79] Mondal B., Gogoi P.K. (2022). Nanoscale Heterostructured Materials Based on Metal Oxides for a Chemiresistive Gas Sensor. ACS Appl. Electron. Mater..

[80] Bhaliya J.D., Shah V.R., Patel G., Deshmukh K. (2023). Recent Advances of MOF-Based Nanoarchitectonics for Chemiresistive Gas Sensors.

[81] Wang L., Cheng Y., Gopalan S., Luo F., Amreen K., Singh R.K., Goel S., Lin Z., Naidu R. (2023). Review and Perspective: Gas Separation and Discrimination Technologies for Current Gas Sensors in Environmental Applications. ACS Sens..

[82] Chen F., Jiang S., Ho H.L., Gao S., Wang Y., Jin W. (2022). Frequency-Division-Multiplexed Multicomponent Gas Sensing with Photothermal Spectroscopy and a Single NIR/MIR Fiber-Optic Gas Cell. Anal. Chem..

[83] Huang X., Sun W., Li Z., Shi J., Zhang N., Zhang Y., Zhai X., Hu X., Zou X. (2022). Hydrogen sulfide gas sensing toward on-site monitoring of chilled meat spoilage based on ratio-type fluorescent probe. Food Chem..

[84] Cai Z., Chen J., Xing S., Zheng D., Guo L. (2021). Highly fluorescent g-C_3_N_4_ nanobelts derived from bulk g-C_3_N_4_ for NO_2_ gas sensing. J. Hazard. Mater..

[85] Chua W.H., Yaacob M.H., Tan C.Y., Ong B.H. (2021). Chemical bath deposition of h-MoO_3_ on optical fibre as room-temperature ammonia gas sensor. Ceram. Int..

[86] Wang Z., Zhang Y., Ren Y., Wang M., Zhang Z., Zhao W., Yan J., Zhai C., Yun J. (2021). NO gas adsorption properties of MoS_2_ from monolayer to trilayer: A first-principles study. Mater. Res. Express.

[87] Tabata H., Matsuyama H., Goto T., Kubo O., Katayama M. (2021). Visible-Light-Activated Response Originating from Carrier-Mobility Modulation of NO_2_Gas Sensors Based on MoS_2_ Monolayers. ACS Nano.

[88] Park S.J., Ha T.J. (2023). MoS_2_ monolayers functionalized with gold nanoparticles using microwave absorption for FET-type NO_2_ gas sensors in ppb-levels. Appl. Surf. Sci..

[89] Wang Y., Zhou Y. (2022). Recent Progress on Anti-Humidity Strategies of Chemiresistive Gas Sensors. Materials.

[90] Shen Y., Tissot A., Serre C. (2022). Recent progress on MOF-based optical sensors for VOC sensing. Chem. Sci..

[91] Wei H., Zhang H., Song B., Yuan K., Xiao H., Cao Y., Cao Q. (2023). Metal–Organic Framework (MOF) Derivatives as Promising Chemiresistive Gas Sensing Materials: A Review. Int. J. Environ. Res. Public Health.

[92] Kidanemariam A., Park J. (2021). Metal-organic framework based on Co and 4,4′-dimethylenebiphenyl diphosphonic acid as an efficient methylene blue adsorbent. J. Ind. Eng. Chem..

[93] Lee J.H., Nguyen T.T.T., Nguyen L.H.T., Phan T.B., Kim S.S., Doan T.L.H. (2021). Functionalization of zirconium-based metal–organic frameworks for gas sensing applications. J. Hazard. Mater..

[94] Hong M., Jo W., Jo S.A., Jin H., Kim M., Lee C.Y., Park Y.D. (2024). Enhanced Gas Sensing Characteristics of a Polythiophene Gas Sensor Blended with UiO-66 via Ligand Functionalization. Adv. Electron. Mater..

[95] Zhang J., Liu J., Liu Y., Li G., Guo J., Zhang J., Zhao Q., Che J., Li L., Gao J. (2023). Design engineering of MOF-derived ZnO porous nanofibers functionalized with Pt clusters: Significantly improved acetone sensing properties. Sens. Actuators B Chem..

[96] Yang D.H., Nguyen T.T.T., Navale S.T., Nguyen L.H.T., Dang Y.T., Mai N.X.D., Phan T.B., Kim J.Y., Doan T.L.H., Kim S.S. (2022). Novel amine-functionalized zinc-based metal-organic framework for low-temperature chemiresistive hydrogen sensing. Sens. Actuators B Chem..

[97] Wu M., Ma Z., Fan Y., Wu Y., An Z., Zhao H., Liu Y., Xu J., Design M. (2022). Sensing Performance and Mechanism of Anhydrous Hydrogen Fluoride Gas Sensor Based on Amino-Functionalized MIL-101(Cr) for New Energy Vehicles. Coatings.

[98] Wang X., Kong D., Li X., Xie K. (2023). MOF-derived hierarchical hollow Fe_2_O_3_ nanobox functionalized with Ru doping for superior H_2_S sensing, Colloids Surfaces A Physicochem. Colloids Surfaces A Physicochem. Eng. Asp..

[99] Park C., Woo J., Jeon M., Baek J.W., Shin E., Kim J., Park S., Kim I.D. (2025). Dual-MOF-Layered Films via Solution Shearing Approach: A Versatile Platform for Tunable Chemiresistive Sensors. ACS Nano.

[100] Cho S., Park C., Jeon M., Lee J.H., Kwon O., Seong S., Kim J., Kim I.D., Moon H.R. (2022). Interface-Sensitized Chemiresistor: Integrated Conductive and Porous Metal-Organic Frameworks. Chem. Eng. J..

[101] Hassan M.H., Haikal R.R., Hashem T., Rinck J., Koeniger F., Thissen P., Hei S.S., Wöll C., Alkordi M.H., Conductive E. (2019). Electrically Conductive, Monolithic Metal–Organic Framework–Graphene (MOF@G) Composite Coatings. ACS Appl. Mater. Interfaces.

[102] Arul C., Moulaee K., Donato N., Iannazzo D., Lavanya N., Neri G., Sekar C. (2021). Temperature modulated Cu-MOF based gas sensor with dual selectivity to acetone and NO2 at low operating temperatures. Sens. Actuators B Chem..

[103] Ren X., Xu Z., Liu D., Li Y., Zhang Z., Tang Z. (2022). Conductometric NO_2_ gas sensors based on MOF-derived porous ZnO nanoparticles. Sens. Actuators B Chem..

[104] Majhi S.M., Kim J.Y., Mirzaei A., Surya S.G., Kim H.W., Kim S.S. (2024). MOF-derived SnO_2_ nanoparticles for realization of ultrasensitive and highly selective NO_2_ gas sensing. Sens. Actuators B Chem..

[105] Zhang S., Zhao Z., Jia L., Guo X., Yang R., Deng Q., Zhang D. (2024). Ultrahigh sensitive and selectivity NO_2_ gas sensors based on Sn-MOF derivates at low temperature. Sens. Actuators B Chem..

[106] Yang C.R., Cheng P.W., Tseng S.F. (2023). Highly responsive and selective NO_2_ gas sensors based on titanium metal organic framework (Ti-MOF) with pyromellitic acid. Sens. Actuators A Phys..

[107] Bag A., Kumar M., Moon D.B., Hanif A., Sultan M.J., Yoon D.H., Lee N.E. (2021). A room-temperature operable and stretchable NO_2_ gas sensor composed of reduced graphene oxide anchored with MOF-derived ZnFe_2_O_4_ hollow octahedron. Sens. Actuators B Chem..

[108] Small L.J., Vornholt S.M., Percival S.J., Meyerson M.L., Schindelholz M.E., Chapman K.W., Nenoff T.M. (2023). Impedance-Based Detection of NO_2_ Using Ni-MOF-74: Influence of Competitive Gas Adsorption. ACS Appl. Mater. Interfaces.

[109] Begi A.N., Hussain S., Amu-Darko J.N.O., Shah S., Junhao W., Zhang X., Yusuf K., Manavalan R.K., Qiao G., Liu G. (2024). Low-concentration H_2_S gas sensors based on MOF-derived Co_3_O_4_ nanomaterials. Sens. Actuators A Phys..

[110] Zhou Q., Yang L., Kan Z., Lyu J., Wang M.X., Dong B., Bai X., Chang Z., Song H., Xu L. (2022). Diverse scenarios selective perception of H_2_S via cobalt sensitized MOF filter membrane coated Three-Dimensional metal oxide sensor. Chem. Eng. J..

[111] Chen X., Liang R., Qin C., Ye Z., Zhu L. (2022). Regulating Co-MOF array films to construct Co_3_O_4_ in-situ sensors for ultrasensitive and highly selective triethylamine detection. Sens. Actuators B Chem..

[112] Yan P., Li X., Ma D., Li L., Lan Y., Li Z., Lu X., Yang M., Liang F. (2022). A cobalt-based MOF with the synergistic effect of size sieving and multi-functional sites for selective gas adsorption. J. Solid State Chem..

[113] Nguyen D.K., Lee J.H., Nguyen T.B., Le Hoang Doan T., Phan B.T., Mirzaei A., Kim H.W., Kim S.S. (2020). Realization of selective CO detection by Ni-incorporated metal-organic frameworks. Sens. Actuators B Chem..

[114] Wang X., Xu X., Zhou T., Zhang T. (2024). Nanoscale MOF-74-based QCM gas sensor for CO_2_ detection at room temperature. Sens. Actuators B Chem..

[115] Ingle N., Sayyad P., Deshmukh M., Bodkhe G., Mahadik M., Al-Gahouari T., Shirsat S., Shirsat M.D. (2021). A chemiresistive gas sensor for sensitive detection of SO_2_ employing Ni-MOF modified –OH-SWNTs and –OH-MWNTs. Appl. Phys. A Mater. Sci. Process..

[116] López-Cervantes V.B., Kim D.W., Obeso J.L., Martínez-Ahumada E., Amador-Sánchez Y.A., Sánchez-González E., Leyva C., Hong C.S., Ibarra I.A., Solis-Ibarra D. (2023). Detection of SO_2_ using a chemically stable Ni(ii)-MOF. Nanoscale.

[117] Zhai Z., Wang J., Sun Y., Hao X., Niu B., Xie H., Li C. (2023). MOFs/nanofiber-based capacitive gas sensors for the highly selective and sensitive sensing of trace SO_2_. Appl. Surf. Sci..

[118] Brandt P., Nuhnen A., Öztürk S., Kurt G., Liang J., Janiak C. (2021). Comparative Evaluation of Different MOF and Non-MOF Porous Materials for SO_2_ Adsorption and Separation Showing the Importance of Small Pore Diameters for Low-Pressure Uptake. Adv. Sustain. Syst..

[119] Zhou L.J., Zhang X.X., Zhang W.Y. (2021). Sulfur dioxide sensing properties of MOF-derived ZnFe_2_O_4_ functionalized with reduced graphene oxide at room temperature. Rare Met..

[120] Carrasco S., Martínez M.L., Amador-Sánchez Y.A., Cervantes V.B.L., Sánchez-González E., Portillo-Vélez N.S., Peralta R.A., Celaya C.A., Guzmán-Vargas A., Horcajada P. (2025). (Hf)PCN-224(Co) as an efficient ppm-level sensor for toxic SO_2_. Mater. Today Adv..

[121] Ren Y.B., Xu H.Y., Gang S.Q., Gao Y.J., Jing X., Du J.L. (2022). An ultra-stable Zr(IV)-MOF for highly efficient capture of SO_2_ from SO_2_/CO_2_ and SO_2_/CH4 mixtures. Chem. Eng. J..

[122] López-Olvera A., Zárate J.A., Martínez-Ahumada E., Fan D., Díaz-Ramírez M.L., Sáenz-Cavazos P.A., Martis V., Williams D.R., Sánchez-González E., Maurin G. (2021). SO_2_ Capture by Two Aluminum-Based MOFs: Rigid-like MIL-53(Al)-TDC versus Breathing MIL-53(Al)-BDC. ACS Appl. Mater. Interfaces.

[123] Zhai Z., Zhang X., Wang J., Li H., Sun Y., Hao X., Qin Y., Niu B., Li C. (2022). Washable and flexible gas sensor based on UiO-66-NH_2_ nanofibers membrane for highly detecting SO_2_. Chem. Eng. J..

[124] Obeso J.L., Martínez-Ahumada E., López-Olvera A., Ortiz-Landeros J., Lara-García H.A., Balmaseda J., López-Morales S., Sánchez-González E., Solis-Ibarra D., Leyva C. (2023). MOF-303 as an Effective Adsorbent to Clean CH_4_: SO_2_ Capture and Detection. ACS Appl. Energy Mater..

[125] Hu L., Hu M., Zhang M., Wang W., Jiang L., Wu W., Lin D., Yang K. (2025). A novel amino-functionalized Bio-MOF for trace SO_2_ adsorption under dry and humid conditions. Sep. Purif. Technol..

[126] Qin P., Day B.A., Okur S., Li C., Chandresh A., Wilmer C.E., Heinke L. (2022). VOC Mixture Sensing with a MOF Film Sensor Array: Detection and Discrimination of Xylene Isomers and Their Ternary Blends. ACS Sens..

[127] Mousavi S., Zeinali S. (2022). VOC s detection using resistive gas nanosensor based on MIL-101(Cr) as a metal organic framework. Sens. Actuators A Phys..

[128] Xu H., Zhong H., Hu J., Rong X., Zhang W., Wang Y., Li S., Li G., Wang D. (2024). Facile engineering of metal–organic framework derived SnO_2_@NiO core–shell nanocomposites based gas sensor toward superior VOCs sensing performance. Chem. Eng. J..

[129] Wang S., Pirzada T., Xie W., Barbieri E., Hossain O., Opperman C.H., Pal L., Wei Q., Parsons G.N., Khan S.A. (2022). Creating hierarchically porous banana paper-metal organic framework (MOF) composites with multifunctionality. Appl. Mater. Today.

[130] Gao L., Kou D., Ma W., Zhang S. (2023). Biomimetic Metal-Organic Framework-Based Photonic Crystal Sensor for Highly Sensitive Visual Detection and Effective Discrimination of Benzene Vapor. ACS Appl. Mater. Interfaces.

[131] Ma X., Wu J., Jiang L., Wang M., Deng G., Qu S., Chen K. (2021). On-chip integration of a metal-organic framework nanomaterial on a SiO_2_waveguide for sensitive VOC sensing. Lab Chip.

[132] Hu J., Chen S., Liu Z., Li J.R., Huang J.H., Jiang Z., Ou W., Liao W.M., Lu J., He J. (2023). MOF-based colorimetric sensor for rapid and visual readout of trace acetylene. J. Mater. Chem. A.

[133] Feng L., Musto C.J., Kemling J.W., Lim S.H., Suslick K.S. (2010). A colorimetric sensor array for identification of toxic gases below permissible exposure limits. Chem. Commun..

[134] Fan L., Zhang J., Zhao Y., Sun C., Li W., Chang Z. (2024). A robust Eu-MOF as a multi-functional fluorescence sensor for detection of benzaldehyde, Hg2+, and Cr2O72-/CrO42-. Microchem. J..

[135] López-Cervantes V.B., López-Olvera A., Obeso J.L., Torres I.K., Martínez-Ahumada E., Carmona-Monroy P., Sánchez-González E., Solís-Ibarra D., Lima E., Jangodaz E. (2024). Robust Co(II)-Based Metal-Organic Framework for the Efficient Uptake and Selective Detection of SO_2_. Chem. Mater..

[136] Jing C., Wang Y., Song X., Li X., Kou M., Zhang G., Dou W., Liu W. (2023). Dual-Fluorophore and Dual-Site Multifunctional Fluorescence Sensor for Visualizing the Metabolic Process of GHS to SO_2_ and the SO_2_ Toxicological Mechanism by Two-Photon Imaging. Anal. Chem..

[137] Peng H., Kong S., Deng X., Deng Q., Qi F., Liu C., Tang R. (2023). Development of a Ratiometric Fluorescent Probe with Zero Cross-Talk for the Detection of SO_2_ Derivatives in Foods and Live Cells. J. Agric. Food Chem..

[138] Obeso J.L., López-Cervantes V.B., Flores C.V., Martínez A., Amador-Sánchez Y.A., Portillo-Velez N.S., Lara-García H.A., Leyva C., Solis-Ibarra D., Peralta R.A. (2024). CYCU-3: An Al(iii)-based MOF for SO_2_ capture and detection. Dalt. Trans..

[139] Zhao W., Obeso J.L., López-Cervantes V.B., Bahri M., Sánchez-González E., Amador-Sánchez Y.A., Ren J., Browning N.D., Peralta R.A., Barcaro G. (2025). Achieving Sub-ppm Sensitivity in SO 2 Detection with a Chemically Stable Covalent Organic Framework. Angew. Chemie.

[140] Che S., Shou Q., Fan Y., Peng X., Zhou C.S., Fu H., She Y. (2022). Fluorescent Ionic Liquid Membranes Based on Coumarin for the Real-Time and Visual Detection of Gaseous SO_2_. ACS Sustain. Chem. Eng..

[141] Ji Y., Ma T., Yang X., Wang R., Wang K., Liu X., Wang X., Jin Q. (2025). Innovative SIFSIX-1-Cu-based microwave sensor with directionally ordered interconnected nanochannels: Revolutionizing high-performance SO_2_ detection. J. Hazard. Mater..

[142] Deng S., Liu J., Han D., Yang X., Liu H., Zhang C., Blecker C. (2024). Synchronous fluorescence detection of nitrite in meat products based on dual-emitting dye@MOF and its portable hydrogel test kit. J. Hazard. Mater..

[143] Kajal N., Gautam S. (2022). Efficient nitro-aromatic sensor via highly luminescent Zn-based metal-organic frameworks: Nitro-aromatic sensor via Zn-based metal-organic frameworks. Chem. Eng. J. Adv..

[144] Hurlock M.J., Christian M.S., Small L.J., Percival S.J., Rademacher D.X., Schindelholz M.E., Nenoff T.M. (2024). Exceptional Electrical Detection of Trace NO_2_ via Mixed Metal MOF-on-MOF Film-Based Sensors. ACS Appl. Mater. Interfaces.

[145] Zhang J., Hu E., Liu F., Li H., Xia T. (2021). Growth of robust metal-organic framework films by spontaneous oxidation of a metal substrate for NO_2_ sensing. Mater. Chem. Front..

[146] Mandal W., Majumder D., Fajal S., Let S., Shirolkar M.M., Ghosh S.K. (2023). Post engineering of a chemically stable MOF for selective and sensitive sensing of nitric oxide. Mol. Syst. Des. Eng..

[147] Xu R., Sun B., Ji W., Sun J., Li P., Ren Z., Jing L. (2024). Construction of a CoNiHHTP MOF/PHI Z-Scheme Heterojunction for ppb Level NO_2_ Photoelectric Sensing with 405 nm Irradiation at RT. ACS Sensors.

[148] Liang M., Gao Y., Sun X., Kong R.M., Xia L., Qu F. (2024). Metal-organic framework-based ratiometric point-of-care testing for quantitative visual detection of nitrite. J. Hazard. Mater..

[149] Ambaye A.D., Muchindu M., Jijana A., Mishra S., Nxumalo E. (2023). Screen-printed electrode system based on carbon black/copper-organic framework hybrid nanocomposites for the electrochemical detection of nitrite. Mater. Today Commun..

[150] Zaimbashi R., Salarizadeh N., Askari M.B. (2024). Electrochemical Oxidation of Glutathione in the Presence of Tryptophan at Carbon Paste Electrode Modified with Ni-Zn-Metal-Organic Frameworks/Graphene Oxide and Ferrocene Derivative. J. Electrochem. Soc..

[151] Feng Y., Luan S., Yi J., Zhang Y., Li X., Lv S., Cong Y. (2024). A novel electrochemical sensor for simultaneous determination of 2,4-dichlorophenol and 3-chlorophenol. Environ. Sci. Nano.

[152] Dey B., Sarkhel G., Choudhury A. (2023). Facile synthesis of copper MOF/carbon nanofiber nanocomposite paper for electrochemical detection of toxic 4-nitrophenol. J. Macromol. Sci. Part A.

[153] Guan H., Chen Y., Xing K., Liu Q. (2024). Electrochemical sensor based on Fe3O4@Au/MOF-P2W17V composite modified glassy carbon electrode for food nitrite detection. J. Food Compos. Anal..

[154] More M.S., Bodkhe G.A., Singh F., Kim M., Shirsat M.D. (2023). Metal–organic framework-reduced graphene oxide (Zn-BDC@rGO) composite for selective discrimination among ammonia, carbon monoxide, and sulfur dioxide. Appl. Phys. A Mater. Sci. Process..

[155] Wu Y., Li W., Chang Y., Gao Y., Wang F., Li H., French P.J., Lee Y.K., Akbar S.A., Siddiqui A.M.U. (2025). NO Detection on Exposed Fe-N4 Sites Deposited on Nanometer-Sized Cu-Hemin MOFs Coated on Reduced Graphene Oxide at Room Temperature. ACS Appl. Nano Mater..

[156] Yang C.R., Huang J.G., Huang M.J., Shen H.Y., Tseng S.F. (2024). High-performance NO_2_ gas sensors based on vanadium metal organic frameworks (V-MOFs) on flexible graphene electrodes. J. Alloys Compd..

[157] Huang H., Chen Y., Chen Z., Chen J., Hu Y., Zhu J.J. (2021). Electrochemical sensor based on Ce-MOF/carbon nanotube composite for the simultaneous discrimination of hydroquinone and catechol. J. Hazard. Mater..

[158] Wang S., Xue Y., Yu Z., Huang F., Jin Y. (2023). Layered 2D MOF nanosheets grown on CNTs substrates for efficient nitrite sensing. Mater. Today Chem..

[159] Hassan K., Tran A.T.T., Jalil M.A., Tung T.T., Nine M.J., Losic D. (2025). Machine Learning-Enhanced Chemiresistive Sensors for Ultra-Sensitive Detection of Methanol Adulteration in Alcoholic Beverages. ACS Sens..

[160] More M.S., Bodkhe G.A., Singh F., Dole B.N., Tsai M.L., Hianik T., Shirsat M.D. (2024). Hydrogen sulfide chemiresistive sensor based on swift heavy ion irradiated cerium-based metal–organic framework/graphene oxide composite. Synth. Met..

[161] Zeng W., Luo S., Rong D., Li Y., Fei Y., Zhao M., Huang L. (2025). The NH4+ Electrochemical Sensor with the 3D-2D Electron Transfer Channel Based on ZIF-8/rGO Composite Frameworks. J. Phys. Chem. C.

[162] Ali A., Alzamly A., Greish Y.E., Bakiro M., Nguyen H.L., Mahmoud S.T. (2021). A highly sensitive and flexible metal-organic framework polymer-based H_2_S gas sensor. ACS Omega.

[163] Ali A., Altakroori H.H.D., Greish Y.E., Alzamly A., Siddig L.A., Qamhieh N., Mahmoud S.T. (2022). Flexible Cu_3_(HHTP)2 MOF Membranes for Gas Sensing Application at Room Temperature. Nanomaterials.

[164] Ahmadipour M., Peterson G.W., Montazami R. (2025). Smart Textile: Functionalization and Electrohydrodynamic-Jet Printing of UiO-66-NH2 Metal-Organic Frameworks for Gas-Sensing Applications. ACS Appl. Mater. Interfaces.

[165] Hizam S.M.M., Saheed M.S.M. (2023). Facile Electrochemical Approach Based on Hydrogen-Bonded MOFs-Derived Tungsten Ethoxide/Polypyrrole-Reduced GO Nanocrystal for ppb Level Ammonium Ions Detection. Chemosensors.

[166] Huang B., Li Y., Zeng W. (2021). Application of metal-organic framework-based composites for gas sensing and effects of synthesis strategies on gas-sensitive performance. Chemosensors.

[167] Maji B., Dash P. (2024). Investigation into the enhanced gas sensing performance for CH4: Comparative study of MOF-derived and traditionally synthesized ZnCo_2_O_4_ flower based composite. Sens. Actuators B Chem..

[168] Sahoo S.J., Das A., Maji B., Goutam U.K., Dash P. (2024). Effect of a Ni-Substituted Cu-MOF Precursor toward Efficient NO_2_ Gas Detection: A Comprehensive Comparative Gas-Sensing Study of MOF-Derived and Traditionally Synthesized CuO/NiO-Based Nanocomposites. ACS Appl. Electron. Mater..

[169] Xue S., Cao S., Huang Z., Yang D., Zhang G. (2021). Improving gas-sensing performance based on mos nanomaterials: A review. Materials.

[170] Veríssimo M.I.S. (2024). A critical review of the analytical performance of the most recent MOS-based gas sensors for indoor air quality monitoring of WHO priority pollutants. TrAC—Trends Anal. Chem..

[171] Zeama M., Alhaji A., Serre C., Shekhah O., Eddaoudi M. (2025). A Metal–Organic Framework Based Gas Sensor for Real-Time Formaldehyde Detection: Metal(III/IV) Pyrazole Carboxylates as a Case Study. Chem. Mater..

[172] Sharma A., Karuppasamy K., Vikraman D., Cho Y., Adaikalam K., Korvink J.G., Kim H.S., Sharma B. (2022). Metal Organic Framework-Derived ZnO@GC Nanoarchitecture as an Effective Hydrogen Gas Sensor with Improved Selectivity and Gas Response. ACS Appl. Mater. Interfaces.

[173] Li S., Zhang Y., Han L., Li X., Xu Y. (2021). Hierarchical kiwifruit-like ZnO/ZnFe_2_O_4_ heterostructure for high-sensitive triethylamine gaseous sensor. Sens. Actuators B Chem..

[174] He Z., Hu J., Zhong J., Long Y., Shen J., Chen S., Ou W., Liu Q., Lu J., Lou Z. (2025). Plasmonic MOF for Highly Selective SERS Sensing of Trace Mercury (II) in Complex Matrices. Small.

[175] Zhang B., Cao X., Wen J., Guo S., Duan X., Zhang X.M. (2024). Integration of Plasmonic materials with MOFs/MOF-derived materials for Photocatalysis. Coord. Chem. Rev..

[176] Phan-Quang G.C., Yang N., Lee H.K., Sim H.Y.F., Koh C.S.L., Kao Y.C., Wong Z.C., Tan E.K.M., Miao Y.E., Fan W. (2019). Tracking airborne molecules from afar: Three-dimensional metal-organic framework-surface-enhanced raman scattering platform for stand-off and real-time atmospheric monitoring. ACS Nano.

[177] Zheng G., Pastoriza-Santos I., Pérez-Juste J., Liz-Marzán L.M. (2021). Plasmonic metal-organic frameworks. SmartMat.

[178] Lafuente M., De Marchi S., Urbiztondo M., Pastoriza-Santos I., Pérez-Juste I., Santamaría J., Mallada R., Pina M. (2021). Plasmonic MOF Thin Films with Raman Internal Standard for Fast and Ultrasensitive SERS Detection of Chemical Warfare Agents in Ambient Air. ACS Sens..

[179] Koh C.S.L., Sim H.Y.F., Leong S.X., Boong S.K., Chong C., Ling X.Y. (2021). Plasmonic Nanoparticle-Metal-Organic Framework (NP-MOF) Nanohybrid Platforms for Emerging Plasmonic Applications. ACS Mater. Lett..

[180] Praveenchandar J., Vetrithangam D., Kaliappan S., Karthick M., Pegada N.K., Patil P.P., Rao S.G., Umar S. (2022). IoT-Based Harmful Toxic Gases Monitoring and Fault Detection on the Sensor Dataset Using Deep Learning Techniques. Sci. Program..

[181] Narkhede P., Walambe R., Mandaokar S., Chandel P., Kotecha K., Ghinea G. (2021). Gas detection and identification using multimodal artificial intelligence based sensor fusion. Appl. Syst. Innov..

[182] Hou L., Xu X., Zhong Z., Tian F., Wang L., Xu Y. (2024). Bimetallic MOF-Based Sensor for Highly Sensitive Detection of Ammonia Gases. ACS Appl. Mater. Interfaces.

[183] Ding J., Wang Q., Liu X., Li S., Li H. (2024). Ultrasensitive detection of hazardous gas at room temperature enabled by MOF@MXene 0D-2D heterostructure. J. Hazard. Mater..

[184] Amu-Darko J.N.O., Hussain S., Agyekum E.A., Begi A.N., Shah S., Yusuf K., Manavalan R.K., Qiao G., Liu G. (2024). Low-Temperature NO_2_ Gas-Sensing System Based on Metal-Organic Framework-Derived In_2_O_3_ Structures and Advanced Machine Learning Techniques. Inorg. Chem..

[185] Mumtaz F., Zhang B., Subramaniyam N., Roman M., Holtmann P., Hungund A.P., O’Malley R., Spudich T.M., Davis M., Gerald R.E. (2024). Miniature Optical Fiber Fabry-Perot Interferometer Based on a Single-Crystal Metal-Organic Framework for the Detection and Quantification of Benzene and Ethanol at Low Concentrations in Nitrogen Gas. ACS Appl. Mater. Interfaces.

[186] Song Z., Bing Y., Cao S., Jiang H., Xu X., Zhou T., Zhang T. (2025). MEMS gas sensor based on ZnCu-based oxide semiconductors for H 2 S detection. IEEE Electron Device Lett..

[187] Amu-Darko J.N.O., Hussain S., Zhang X., Alothman A.A., Ouladsmane M., Nazir M.T., Qiao G., Liu G. (2023). Metal-organic frameworks-derived In_2_O_3_/ZnO porous hollow nanocages for highly sensitive H2S gas sensor. Chemosphere.

[188] Li M., He X., Wu C., Wang L., Zhang X., Gong X., Zeng X., Huang Y. (2024). Deep Learning Enabled SERS Identification of Gaseous Molecules on Flexible Plasmonic MOF Nanowire Films. ACS Sens..

[189] Jang Y.W., Jo J.W., Park S.K., Kim J. (2024). Room-temperature gas sensors based on low-dimensional nanomaterials. J. Mater. Chem. C.

[190] Fan Y., Liu C., Zhao K., Shao T., Chen R., Pan Y., Liu Z., Pan X. (2023). A self-powered flexible sensor based on thermoelectric generation for NO_2_ gas detection. Int. J. Green Energy.

[191] Machín A. (2025). Next-Generation Chemical Sensors: The Convergence of Nanomaterials, Advanced Characterization, and Real-World Applications. Chemosensors.

[192] Wang J., Wang R. (2025). Development of Gas Sensors and Their Applications in Health Safety, Medical Detection, and Diagnosis. Chemosensors.

[193] Stassen I., Burtch N., Talin A., Falcaro P., Allendorf M., Ameloot R. (2017). An updated roadmap for the integration of metal-organic frameworks with electronic devices and chemical sensors. Chem. Soc. Rev..

[194] Ulybyshev D., Yilmaz I., Northern B., Kholodilo V., Rogers M. (2021). Trustworthy Data Analysis and Sensor Data Protection in Cyber-Physical Systems, SAT-CPS 2021—Proc. 2021 ACM Work. Secur. Trust. Cyber-Physical Syst..

[195] Iqbal Z., Zaidi A.A., Javed M., Xin L. (2024). Progress in the Application of MOF-based SERS Sensors in Biomedicine. Res. J. Adv. Eng. Sci..

[196] Dwivedi A.K., Kar N.B., Shivhare A. Cyber Security Threat Modeling of IoT Security Design Patterns. Proceedings of the 2025 International Conference on Intelligent and Cloud Computing.

[197] Akram W., Iqbal S., Abbas Z., Mansoor S., Shah I. (2025). Metal Organic Framework based Humidity Sensing: Stability, performance, and IoT Integration. J. Sci. Adv. Mater. Devices.

[198] Yin W., Huang L., Wang X., Yin W. Research on Intelligent Agricultural Environment Monitoring System Based on LoRa and NB-IoT Technology. Proceedings of the 2024 4th Asian Conference on Innovation in Technology.

[199] Ouyang Q., Rong Y., Xia G., Chen Q., Ma Y., Liu Z. (2025). Integrating Humidity-Resistant and Colorimetric COF-on-MOF Sensors with Artificial Intelligence Assisted Data Analysis for Visualization of Volatile Organic Compounds Sensing. Adv. Sci..

[200] Hui Y., Guo H., Wang M., Peng L., Ren B., Ma Y., Yang W. (2025). Deep machine learning-assisted MOF@COF fluorescence/colorimetric dual-mode intelligent ratiometric sensing platform for sensitive glutathione detection. Talanta.

